# Complete genome sequencing and analysis of a Lancefield group G *Streptococcus dysgalactiae *subsp. *equisimilis *strain causing streptococcal toxic shock syndrome (STSS)

**DOI:** 10.1186/1471-2164-12-17

**Published:** 2011-01-11

**Authors:** Yumi Shimomura, Kayo Okumura, Somay Yamagata Murayama, Junji Yagi, Kimiko Ubukata, Teruo Kirikae, Tohru Miyoshi-Akiyama

**Affiliations:** 1 Department of Infectious Diseases, National Center for Global Health and Medicine, 1-21-1, Toyama, Shinjuku-ku, Tokyo 162-8655, Japan; 2 Graduate School of Infection Control Science, Kitasato University, 5-9-1, Shirokane, Minato-ku, Tokyo 108-8641, Japan; 3 Department of Microbiology and Immunology, Tokyo Women's Medical University, 8-1 Kawada-cho, Shinjuku-ku, Tokyo 162-8666, Japan; 4 Department of Animal and Food Hygiene, Obihiro University of Agriculture and Veterinary Medicine, 2-11 Inada-cho, Obihiro, Hokkaido 080-8555, Japan

## Abstract

**Background:**

*Streptococcus dysgalactiae *subsp. *equisimilis *(SDSE) causes invasive streptococcal infections, including streptococcal toxic shock syndrome (STSS), as does Lancefield group A *Streptococcus pyogenes *(GAS). We sequenced the entire genome of SDSE strain GGS_124 isolated from a patient with STSS.

**Results:**

We found that GGS_124 consisted of a circular genome of 2,106,340 bp. Comparative analyses among bacterial genomes indicated that GGS_124 was most closely related to GAS. GGS_124 and GAS, but not other streptococci, shared a number of virulence factor genes, including genes encoding streptolysin O, NADase, and streptokinase A, distantly related to SIC (DRS), suggesting the importance of these factors in the development of invasive disease. GGS_124 contained 3 prophages, with one containing a virulence factor gene for streptodornase. All 3 prophages were significantly similar to GAS prophages that carry virulence factor genes, indicating that these prophages had transferred these genes between pathogens. SDSE was found to contain a gene encoding a superantigen, streptococcal exotoxin type G, but lacked several genes present in GAS that encode virulence factors, such as other superantigens, cysteine protease *speB*, and hyaluronan synthase operon *hasABC*. Similar to GGS_124, the SDSE strains contained larger numbers of clustered, regularly interspaced, short palindromic repeats (CRISPR) spacers than did GAS, suggesting that horizontal gene transfer via streptococcal phages between SDSE and GAS is somewhat restricted, although they share phage species.

**Conclusion:**

Genome wide comparisons of SDSE with GAS indicate that SDSE is closely and quantitatively related to GAS. SDSE, however, lacks several virulence factors of GAS, including superantigens, SPE-B and the *hasABC *operon. CRISPR spacers may limit the horizontal transfer of phage encoded GAS virulence genes into SDSE. These findings may provide clues for dissecting the pathological roles of the virulence factors in SDSE and GAS that cause STSS.

## Background

Since Lancefield group G streptococci (GGS) have been considered components of the normal flora of the human throat, skin, and genitourinary tract, the contributions of GGS to streptococcal disease have often been overlooked [1]. Over the last decade, however, infections by pathogenic GGS have been reported, including life-threatening invasive β-hemolytic streptococcal disease [1-7], making it necessary to expand our knowledge of the pathogenesis of GGS infections, especially invasive infections. Several species of streptococci can carry group C and G antigens, including *Streptococcus dysgalactiae *subsp. *equisimilis *(SDSE), *S. canis*, *S. dysgalactiae *subsp. *dysgalactiae*, *S. equi *subsp. *equi *(SESE), *S. equi *subsp. *zooepidemicus *(SESZ), and *S. anginosus *group bacteria. SDSE, which consists of Lancefield group G and C bacteria, in a ratio of about 4:1 [3,8,9], has been isolated from patients at higher frequency than other GGS and GCS species. For example, of 313 strains of GCS and GGS isolated from clinical samples in Southern India between 2006 and 2007, 254 (81.1%) were SDSE [9], as were 80% of the 266 invasive non-A and non-B β-hemolytic streptococcal isolates in the USA [3]. The spectrum and clinical courses of SDSE infection, including pharyngitis, cellulitis, infective arthritis, vertebral osteomyelitis, and streptococcal toxic shock syndrome (STSS), show substantial overlap with those of GAS [10-16]. Despite the increased clinical importance of SDSE, however, the entire SDSE genome has not yet been sequenced. Knowledge of its entire genome sequence is fundamental to gain insights into the pathogenicity of SDSE. We describe here the entire genome sequence of a Lancefield group G SDSE strain, GGS_124, which had been isolated from a patient with STSS.

## Results

### Selection of an SDSE isolate for genome sequencing

We chose a clinical isolate of SDSE, strain GGS_124, for genome sequence determination for several reasons. First, GGS_124 belongs to Lancefield group G, to which most clinical isolates of SDSE also belong [3,8,9]. Second, GGS_124 caused STSS in a patient. Third, GGS_124 was the most virulent strain among 8 group G SDSE isolates, as determined by their LD_50 _values in a mouse infection model (Table 1).

**Table 1:** *emm *types and mouse LD_50 _values of 8 SDSE isolates used in this study.

Strain	Origin	Symptom STSS/Non- STSS	**LD**_**50 **_**value** (CFU/head)	*emm *type
GGS_124	human	STSS	2.1 × 10^6^	*stG480.0*
168	human		4.6 × 10^6^	*stG480.0*
GGS_117	human	STSS	5.6 × 10^6^	*stG4974.1*
170	human		5.6 × 10^6^	*stC36.0*
164	human		1.9 × 10^7^	*stG485*
GGS_118	human	STSS	2.0 × 10^7^	*stG67920*
169	human		4.4 × 10^7^	*stG11*
163	human		4.5 × 10^7^	*stG643*

LD_50 _values of the isolates were determined as described in MATERIALS AND METHODS.

### Overview of the SDSE GGS_124 genome sequence

**Figure 1:**
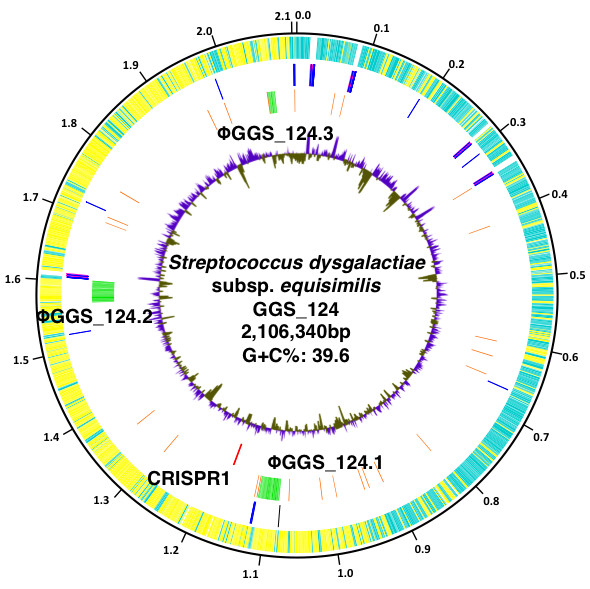
**Circular representation of the genome of *S. dysgalactiae *subsp. *Equisimilis *strain GGS_124**. Circle 1 (outermost circle) indicates the distance from the putative origin of replication. Circle 2 shows annotated CDS encoded on the forward (light blue) and reverse (yellow) chromosomal strands, respectively. The rRNA genes (pink), tRNA genes (blue), and tmRNA gene (black) are shown in circle 3. Prophage (green) and ISs (orange) genes are shown in circle 4. CRISPR (red) is shown in circle 5. Circle 6 (innermost circle) shows the G+C content with greater and less than average (0.40) in purple and brown, respectively.

We found that, similar to other streptococcal genomes, the SDSE GGS_124 genome consists of a single circular chromosome of 2,106,340 bp (Additional file 1) and has a G+C content of 39.6% (Figure 1). Based on its location in the intergenic region upstream of the *dnaA *gene (SDEG_0001), the GC skew, and the clustering of dnaA box motifs, the start point of the SDSE GGS_124 genome was assigned to the putative origin of replication (*oriC*). An AT-rich 13-mer (AGTCTGTTTTTTT), located in the intergenic region upstream of the *dnaA *gene [17], was selected as the starting point for nucleotide numbering. The GGS_124 genome was shown to contain 2095 predicted coding sequences (CDS), which account for 1.83 Mbp (86.9%) of the genome. In addition, this genome was shown to harbor 3 prophage-like elements, designated ΦGGS_124.1, ΦGGS_124.2, and ΦGGS_124.3. Moreover, there were 27 insertion sequence (IS) elements throughout the genome.

Genome sequence homology analysis of GGS_124 with the other 11 sequenced streptococcal species and subspecies showed that GGS_124 was closest in sequence to GAS, with 72% similarity (Additional file 1). GGS_124 was less similar to SESZ and SESE, with 65% and 64% coverage. Although *S. agalactiae *is the closest-relati1ve of SDSE based on 16S rRNA analysis, the *S. agalactiae *strains were less similar to SDSE than GAS based on the genome wide comparison (Additional file 1). In addition, we constructed a phylogenetic tree of all sequenced *Streptococcus *species based on the neighbor-joining method (Additional file 2). Although neighbor-joining methods are less accurate than the other methods such as most likelihood methods, SDSE is clustered with the GAS strains as their closest relative.

**Figure 2:**
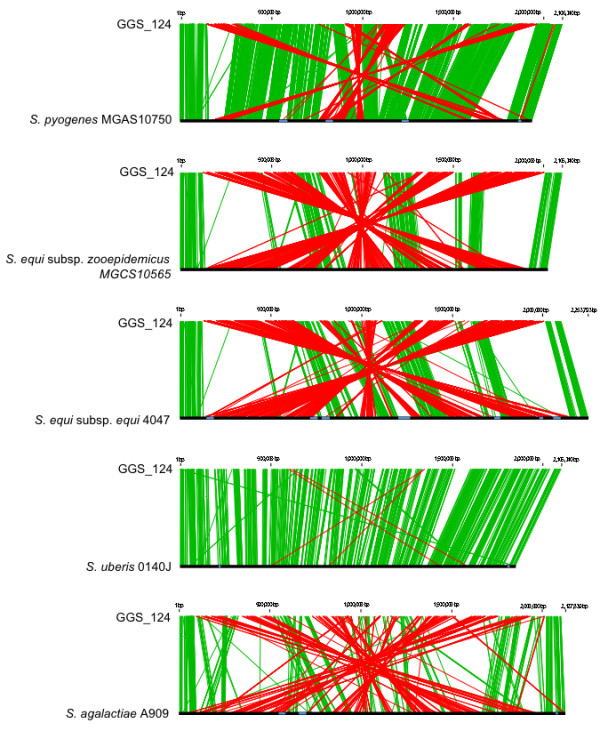
**Genome rearrangement maps of *S. dysgalactiae *subsp. *equisimilis *GGS_124 with five species in the pyogenic group**. Sequences were aligned from the predicted replication origin of each genome. The colored bars separating each genome (red and green) represent similarity matches identified by *in silico *Molecular Cloning. Links shown in green match in the same orientation, while those in red match in the reverse orientation. Prophages are highlighted as pale blue boxes.

We compared the gene organization of GGS_124 with that of GAS by genomic rearrangement analyses (Figure 2 and Additional file 3). GAS could be divided roughly into 2 groups according to the orientation of the genes [18,19]. Therefore, SSI-1 and MGAS315, both of which are M3 serotype strains and have opposite gene orientations from each other, were chosen for the analysis. We found that the GGS_124 genome was organizationally more similar to that of GAS strain MGAS315 than GAS strain SSI-1 (Figure 2). Interestingly, the colinearity of GGS_124 and *S. uberis *genomes was quite remarkable but the percent amino acid identity was lower than that of the GAS strains (Additional file 3). The gene structure of GGS_124 was similar to the structures of GAS strain SSI-1, SESZ strain MGCS10565, and SESE strain 4047, although the GGS_124 genome contains large-scale genomic rearrangements. The GGS_124 genome differed markedly in gene organization from the genome of GBS strain A909.

**Figure 3:**
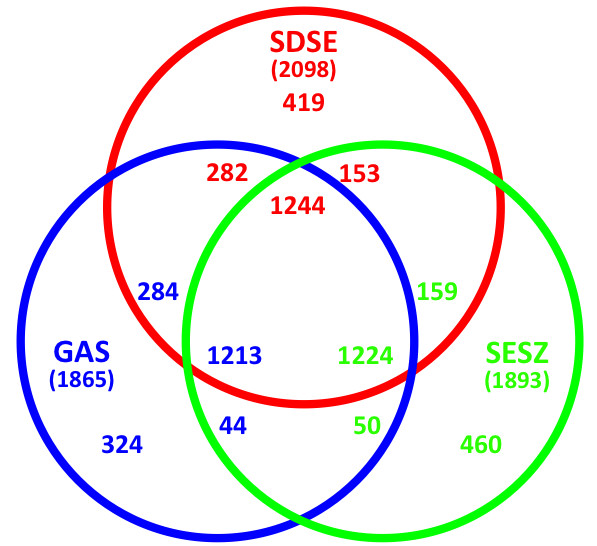
**Venn diagram of gene content comparison among *S. dysgalactiae *subsp. *equisimilis *GGS_124 (SDSE), *S. pyogenes *MGAS315 (GAS) and SESZ MGCS10565**. The inferred proteomes of SDSE, GAS, and SESZ were compared in a pairwise manner with their translated genomes by *in silico *Molecular Cloning and are presented as a Venn diagram. The numbers of products for each section are color coded to match the respective genomes. Genes showing more than 40% identity were considered homologues.

When we compared genes from GGS_124 and two relatively homologous species, GAS (MGAS315) and SESZ (MGCS10565) (Figure 3), we found that these three streptococcal genomes contain more than 1,200 orthologous genes, accounting for 59% of the total CDSs of GGS_124. GGS_124 shares 282 genes with MGAS315 and 153 genes with MGCS10565. Moreover, 71.6% of the genes of GGS_124 were homologous to GAS genes, with 88.5% amino acid identity, whereas 66.5% of GGS_124 genes were homologous to MGCS10565 genes, with 79.9% amino acid identity. These findings indicate that SDSE is closely related to GAS in both nucleotide and amino acid sequences.

We also analyzed the distribution of genes shown to be more homologous to genes derived from bacteria other than GAS (Additional file 4). We found that 299 genes showed higher similarity to genes from *Streptococci *other than GAS and 92 genes showed higher similarity to genes from a genus other than *Streptococcus*. In addition, we identified 11 genes that did not show significant homology to any genes in the databases. These genes were scattered throughout the entire GGS_124 genome, suggesting that they had not been acquired by massive genome recombination.

### Putative prophages and CRISPR/Cas

**Figure 4:**
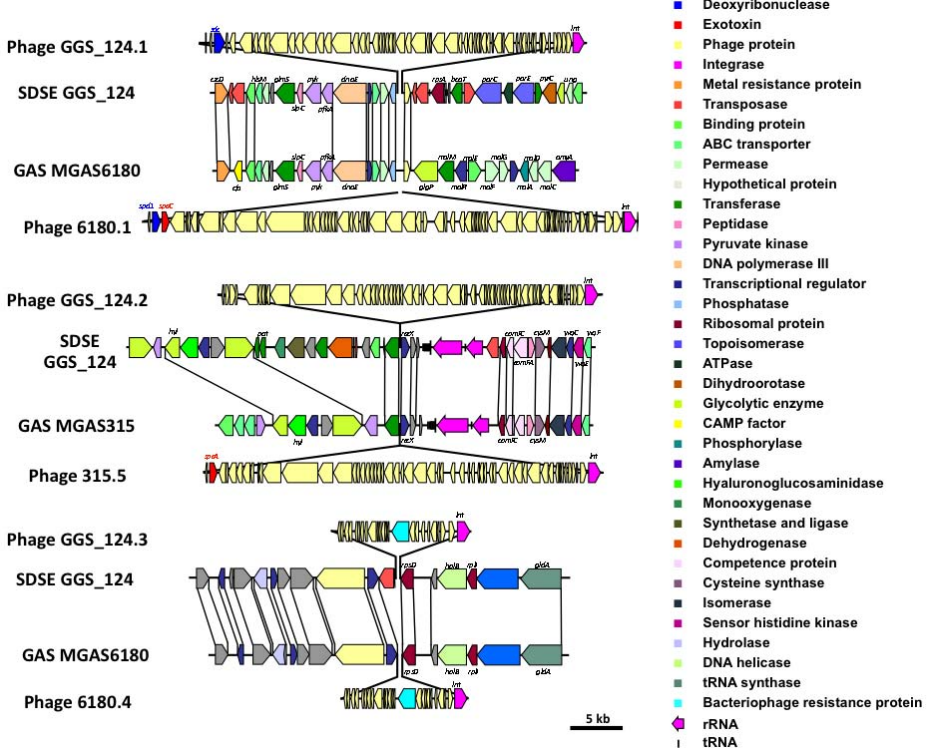
**Prophage elements and the surrounding gene arrangements of GGS_124 and GAS**. The organization of the genes located in the putative prophage regions found in GGS_124 and their insertion points in the genome were compared with those of GAS. Colored boxes between genes indicate level of similarity at the amino acid level (red, ≥90%; orange, 89%-80%; green, 79%-70%).

We found that all three prophage-like elements of GGS_124 were homologous to previously sequenced GAS prophages, and that they were integrated at sites similar to those of GAS strains, with the same upstream and downstream genes (Figure 4).

#### (i) Prophage GGS_124.1

We found that the ΦGGS_124.1 prophage is 35.593 bp in length with a G+C content of 38.04% and carries 60 CDS. Ninety-seven percent of the CDS in ΦGGS_124.1 have homologues, with more than 40% identity to GAS prophages, suggesting that ΦGGS_124.1 is a chimeric phage. This prophage was inserted at the predicted bacteriophage T12*att *site, which has been shown to be a gene that encodes a serine tRNA and is located between the CDS of SDEG_1100 and SDEG_1161 [20]. Six GAS strains, MGAS10394, MGAS315, MGAS5005, MGAS6180, MGAS8232, and SSI-1, have prophage elements: Φ10394.3, which carries *speK *and the streptococcal phospholipase A2 gene (*sla*); Φ315.2, which carries *ssa*; Φ5005.1, which carries *speA*; Φ6180.1, which carries the *speC *and Dnase (*spd*) genes; Φ8232.3, which carries *speL *and *speM*; and SPsP5, which carries *speC*, respectively [18,21-25]. In addition, ΦGGS_124.1 was found to contain a prophage-associated virulence factor gene for deoxyribonuclease (*sdc*).

#### (ii) Prophage GGS_124.2

We found that the ΦGGS_124.2 prophage is 35,814 bp in length, with a G+C content of 38.20% and 61 CDS. Ninety-five percent of the CDS in ΦGGS_124.1 have homology with genes in GAS prophages, making it likely that ΦGGS_124.2 is chimeric phage. The chromosomal phage attachment site (*attB*) and the ΦGGS_124.2 phage-encoded attachment site (*attP*) were not found, but the products of *attP/attB *recombination, *attL *and *attR*, with the same sequences as those of GAS prophages SPsP2 and Φ315.5 were identified. The genome context around the integration site for ΦGGS_124.2 was found to be conserved at the phage integration sites of 4 GAS strains, MGAS10394, MGAS315, SSI-1, and Manfredo, which contain the prophage elements Φ10394.6, carrying *sdn*; Φ315.5, carrying *speA*; SPsP2, carrying *speA*; and phiMan.1, carrying the DNase gene *mf3*, respectively [18,19,21,22]. No known prophage-associated virulence factor genes were found in ΦGGS_124.2.

#### (iii) Prophage GGS_124.3

We also found a prophage remnant, ΦGGS_124.3, which was about 12.6 kb length and homologous to the previously sequenced GAS prophage remnants Φ6180.4 and Φ10270.5 with a nucleotide identity of 73%. ΦGGS_124.3 and the two GAS phage remnants were found to be located between genes encoding a putative transcriptional regulator protein and the 30S ribosomal protein. In strain GGS_124, two truncated transposase proteins, SDEG_2117 and SDEG_2118, were found to be inserted upstream of GGS_124.3. No virulence factor genes are present in GGS_124.3.

**Figure 5:**
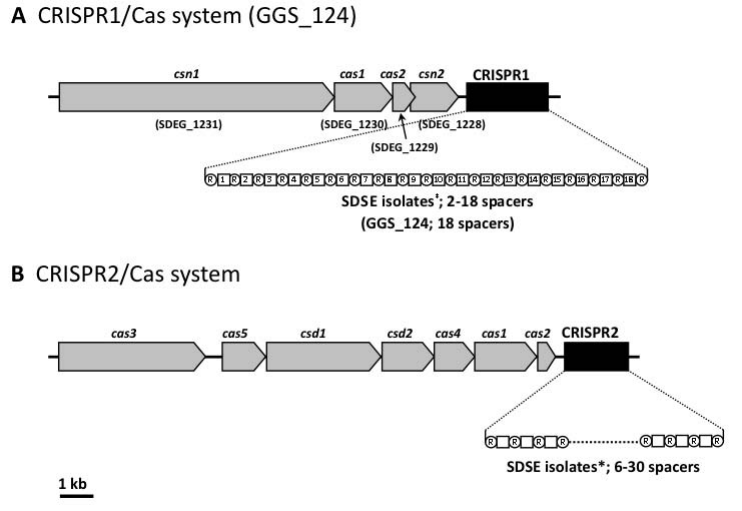
**CRISPR/Cas system structures found in SDSE isolates**. The CRISPR structures of SDSE isolates were analyzed by direct sequencing of PCR-amplified CRISPR regions. The repeat-spacer regions (CRISPR) are enlarged. Open circles marked "R" are direct repeats, whereas the square boxes indicate respective spacers with numbers. GGS_124 contained only CRISPR1, whereas some of the other strains also contained CRISPR2 (Table 2).

Prokaryotes possess a system that mediates resistance to infection by foreign DNA, such as viruses [26,27]. When bacteria are exposed to phages, short fragments derived from phage DNA are integrated into clusters of regularly interspaced short palindromic repeat (CRISPR) regions of the bacterial genome as spacers [27]. CRISPR RNA transcripts and CRISR-associated proteins (Cas), act in complexes to interfere with virus proliferation [26]. This system has also been observed in GAS [20], SESZ [22,28], *S. mutans *[29], and *S. thermophilus *[30]. GGS_124 harbors a CRISPR/Cas system consisting of an array of genes, *can1*, *cas1*, *cas2*, and *csn2*, and CRISPR (Figure 5). The GGS_124 CRISPR has 19 direct repeats of 36 bp each and 18 spacer sequences 30 or 32 bp in length; 6 of these sequences are homologous to GAS prophage sequences, with more than 80% coverage (Additional file 5). When we analyzed the number of CRISPR spacers in an additional 7 SDSE isolates (Table 2), we found that the mean number of CRISPR spacers was higher in SDSE (18.0 ± 3.3 spacers) than in GAS strains (4.0 ± 1.0 spacers; range, 0 to 9) (Table 2). These results suggest that prophage infection of SDSE is somewhat restricted, resulting in a smaller number of virulence factors located in the prophage regions of SDSE. Alternatively, SDSE may be in contact with phages more frequently, with integrated phages having a fitness cost for SDSE.

**Table 2:** Presence of Cas genes and the number of spacers in CRISPR1/Cas and CRISPR2/Cas.

		CRISPR1/Cas			CRISPR2/Cas		
			
Species	Strain	Cas genes	No. of spacers	**Acc. No**.	Cas genes	No. of spacers	Acc. No
*Streptococcus dysgalactiae *subsp. *equisimilis*	GGS_124	+	18	AP010935.1	-	0	-
	168	+	2	AB553332	+	13	AB553333
	GGS_117	+	8	AB553338	+	12	AB553339
	170	+	9	AB553336	+	10	AB553337
	164	+	17	AB553343	+	6	AB553331
	GGS_118	+	8	AB553342	+	13	AB553341
	169	+	7	AB553334	+	30	AB553335
	163	+	3	AB553340	N. D.	N. D.	
*Streptococcus pyogenes*	MGAS8232	-	0	AE009949.1	-	0	0AE009949.1
	MGAS10394	-	0	CP000003.1	-	0	CP000003.1
	MGAS10750	+	0	CP000262.1	+	5	CP000262.1
	Manfredo	-	0	AM295007.1	-	0	AM295007.1
	MGAS10270	+	2	CP000260.1	+	3	CP000260.1
	MGAS315	+	0	AE014074.1	-	0	AE014074.1
	MGAS5005	+	3	CP000017.1	+	4	CP000017.1
	MGAS9429	+	0	CP000259.1	+	7	CP000259.1
	MGAS2096	+	0	CP000261.1	+	6	CP000261.1
	SF370	+	6	AE004092.1	+	3	AE004092.1
	SSI-1	+	0	BA000034.2	-	0	BA000034.2
	MGAS6180	+	4	CP000056.1	+	1	CP000056.1
	NZ131	+	4	CP000829.1	+	5	CP000829.1
*Streptococcus equi *subsp. *zooepidemicus*	MGCS10565	+	17	CP001129.1	+	9	CP001129.1
	H70	-	0	FM204884.1	+	18	FM204884.1
*Streptococcus equi *subsp. *equi*	4047	-	0	FM204883.1	-	0	FM204883.1

N.D.: No amplicon was obtained in PCR analyses.

### Virulence factors encoded by the GGS_124 genome

An analysis of 58 SDSE strains isolated from human infections using targeted microarrays containing 216 GAS virulence genes composed of 70mer oligonucleotides showed that about 50% of the GAS virulence genes represented in the microarray were present in SDSE [31]. Three molecular markers, *speB*, the intergenic region upstream of the *scpG *gene and *virPCR*, have been shown helpful in discriminating between GAS and SDSE [32]. Based on homology analyses with known bacterial virulence factors, such as pore-forming toxins, a superantigen, proteases, FCT-like regions, adhesins, hyaluronidase, and a nuclease, we identified 71 putative virulence factor genes in the GGS_124 genome; their details are shown in Additional file 6. Comparison of the virulence factors in GGS_124 with those of other streptococcal species indicated that the putative virulence factors most similar to those of GGS_124 were found in GAS. In contrast, superantigen, SPE-B and the *has *operon of GAS are not conserved in GGS_124.

#### (i). Pore-forming toxins

GGS_124 has several putative hemolysins, including HlyX (SDEG_0427), HlyIII (SDEG_1015), and HlyA1 (SDEG_1483), which have also been detected in GAS, SESZ, SESE, *S. uberis*, and GBS. GGS_124 also has genes encoding streptolysin S (*sagA*) (SDEG_0705) and its biosynthesis proteins (*sagBCDEFGHI*) (SDEG_0706 to 0713), which are also present in GAS [33], SESZ, and SESE [22,28,34]. In addition, GGS_124 carries a gene for streptolysin O (SLO) (SDEG_2027), which is essential for GAS virulence and is required for the organism to escape from the endosome into the cytosol following invasion of host cells [35].

#### (ii). Superantigen

GGS_124 possesses only one superantigen gene, exotoxin G variant 4 (*spegg4*), which is homologous to GAS streptococcal exotoxin G (SpeG), with 79% amino acid identity (Additional file 6). In a previous analysis of the superantigenic activities of the *spegg4 *product in human peripheral blood mononuclear cells [36], we found that its mitogenic activity was about 1% that of SpeG from GAS. Other genome-encoded superantigen genes for mitogenic exotoxin Z (*smeZ*), which are present in GAS [37], were not found in the GGS_124 genome.

#### (iii) Proteases

We found that a putative proteinase (SDEG_1906) and streptococcal C5a peptidase (*scpB*) (SDEG_0933) [38] were conserved among GGS_124 and 5 closely related species. GGS_124 also has a gene with homology (42% amino acid identity) to exfoliative toxin A of *Staphylococcus aureus *strain Mu50 (SAV1173), which causes staphylococcal scalded skin syndrome [39]. GGS_124 also carries a gene for streptokinase (SDEG_0233), similar to streptokinase A of GAS, with 88% amino acid identity (Additional file 6). This protein complexes with plasminogen to form an activator, which has serine protease activity and cleaves free plasminogen. leading to activation of the zymogen [40]. Strikingly, GGS_124 lacks streptococcal cysteine protease (SpeB), an erythrogenic toxin produced by GAS with cysteine protease activity [41]. The GGS_124 genome lacks approximately 7 kb of the GAS strain MGAS315 sequence, including genes encoding SpeB (SpyM3_1742), the transcriptional regulator RopB (SpyM3_1744), and mitogenic factor 25K precursor (SpyM3_1745). Since several transposase and related genes (SDEG_0212, 0206, 0205, 0201, 0194) are located in the corresponding region, it is highly likely that the region that included *speB *was present in the common ancestor of GAS and SDSE but was not retained by SDSE after speciation.

We found that *speB *was not present in GGS_124, in agreement with the results of a microarray study, which showed that all of the 58 examined strains of group C and G SDSE isolated from patients lacked the *speB *gene [31,32]. We therefore examined whether SDSE strains have protease activity similar to that of SpeB (Additional file 7). We did not detect any SpeB-like protease activity in strains GGS_124 or GGS_118, which had been isolated from two patients with STSS. In contrast, a GAS strain produced a proteinase that was sensitive to E-64, which inhibits cysteine proteases, including SpeB.

#### (iv) FCT-like regions

**Figure 6:**
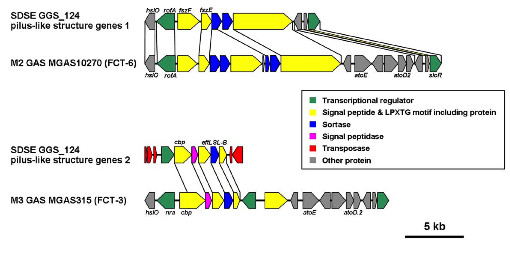
**Organization of genes encoding pilus-like structure proteins in *S. dysgalactiae *subsp. *equisimilis *GGS_124**. The organizations of genes encoding pilus-like structure proteins in GGS_124 were compared with those in GAS and SESZ. Colored boxes between genes indicate similarity at the amino acid level (red, ≥90%; orange, 90%-80%; yellow-green, 79%-70%; green, 69%-60%; purple, 59%-40%; gray, <40%).

Recently, GAS and GBS were shown to express pili, which are synthesized by proteins encoded by genes in FCT regions [1,42,43]. GGS_124 harbors 2 FCT-like regions, which are probable operons expressing different pilus-like structures (Figure 6). One of these contains genes encoding the transcriptional regulator RofA (SDEG_0156), two putative fimbrial structural subunit proteins (SDEG_0157 and SDEG_0158), two sortases (SDEG_0159 and SDEG_0160), and a putative fibronectin binding protein (SDEG_0161). It is similar to the FCT-6 region, which is conserved among M2 GAS, GBS, and SESZ [22,42,44]. The second region contains genes encoding a putative transcriptional regulator (SDEG_1782), a defective collagen binding protein (SDEG_1781), a signal peptidase I (SDEG_1780), a backbone protein (SDEG_1779), and an ancillary protein (SDEG_1778). It is similar to the FCT-3 region, which was found in M3, M5, M18, and M49 GAS [43].

#### (v) Adhesins

GGS_124 possesses genes that encode putative adhesion proteins, including proteins similar to putative fibronectin binding proteins (SDEG_0161, 1263, and 1984), pullulanase (SDEG_0237), phosphopyruvate hydratase (SDEG_0704), laminin binding protein (SDEG_0935), internalin protein (SDEG_1372), and collagen binding protein (SDEG_1781), all of which bind to the extracellular matrix (Additional file 6). SGGS_124 also possesses genes encoding immunoglobulin G binding protein (SDEG_1358) [45] and multifunctional streptococcal plasmin receptor (Plr)/streptococcal surface dehydrogenase (SDH)/glyceraldehyde-3-phosphate dehydrogenase (GAPDH), which binds to complement component C5a (SDEG_1936) [46] (Additional file 6), although the product of SDEG_1936 lacks a signal peptide.

#### (vi) Hyaluronan capsule synthesis

Hyaluronic acid (HA), synthesized *via *the *hasABC *operon, is considered a pleiotropic virulence factor involved in many aspects of GAS infection [47]. GGS_124, however, does not contain an *hasABC *operon, in contrast to the genomes of GAS, SESZ, SESE, and *S. uberis*. Rather, GGS_124 possesses only one gene, encoding glycosyl transferase (SDEG_0628), which shows a low level of similarity to *hasA *of GAS (20% amino acid similarity). Although one SDSE strain has been shown to possess a hyaluronan synthase (AF023876.1), very similar to the product of *hasA *[48], GAS gene microarray analysis of 58 SDSE strains isolated from human infections showed that all harbored only hasC [31]. We found that GGS_124 also harbors only *hasC *(SDEG_1980) (Additional file 6), making it unlikely that SDSE produces HA *via *the *hasABC *operon.

#### (vii) Hyaluronidase

GGS_124 possesses a gene in a non-prophage region of the genome that encodes a putative hyaluronidase (SDEG_0654), with 66% identity to *hyl*B in SESZ (Additional file 6). Hyaluronidase in GAS is thought to contribute to the spread of bacteria in tissues and may allow GAS to utilize host hyaluronic acid or its own capsule as an energy source [49]. The hyaluronidase in GGS_124 may have a function similar to that in GAS.

#### (viii) Nucleases

GGS_124 possesses 5 genes that encode putative nucleases with a secretion signal peptide: genome-encoded streptodornase (SDEG_0541), extracellular nuclease (SDEG_0714), DNA-entry nuclease (SDEG_0732), cell surface 5'-nucleotidase (SDEG_0825), and prophage-associated deoxyribonuclease (SDEG_1103), all of which are predicted to code for a secretion signal peptide. Two of them, SDEG_0714 and SDEG_0825, code for potential cell wall anchor motifs, LPKAG and LPMAG, respectively (Additional file 6). The putative streptodornase SDEG_0541 and DNA-entry nuclease SDEG_0732 are homologous to phage-encoded extracellular streptodornase D Sda1 of GAS (PHA01790) [50] and DNA-entry nuclease EndA of *S. pneumoniae *TIGR4 (SP_1964) [51], respectively (Additional file 6). Sda1 and EndA have been found to degrade neutrophil extracellular traps (NETs) [50,51], which are composed of granule proteins and chromatin released by neutrophils and can catch and kill surrounding bacteria [52]. The putative extracellular DNase SDEG_0714 is similar to M1 GAS cell-wall-located DNase SpnA (Spy0747), which has been reported important for virulence, *e.g*., dispersion in host tissue [53] (Additional file 6).

#### (ix) Other virulence factors

GGS_124 possesses genes encoding the multifunctional M protein (*stg480.0*) (SDEG_0230). The M protein of GAS shows antiphagocytic and adhesin activities, whereas the adhesion function of the GGS M protein may be due to a collagen binding motif [54,55]. Since the product of *Stg480.0 *lacks this motif, the M protein of GGS_124 may not act as an adhesin.

Streptococcal inhibitor of complement (SIC) and distantly related to SIC (DRS) are some extent of homology. DRS binds complement factors but does not inhibit complement mediated cell lysis [56,57], whereas SIC inhibits complement mediated cell lysis [58]. GGS_124 harbors a putative DRS gene (SDEG_0932), which consists of a signal sequence, two repeat regions, and a proline-rich region typical of DRS, and is homologous to the Drs12.04 protein of GAS strain *emm12 *with 48% amino acid identity [59] (Additional file 6).

The GGS_124 genome harbors a gene encoding a collagen-like protein (SDEG_1113), similar to the collagen-like repeat phage protein of SESE 4047 (SEQ_0837), with 41% amino acid identity. Streptococcal collagen-like proteins (Scl) are cell-surface molecules of GAS with domains containing tracks of repeated Gly-Xaa-Yaa sequences that form a mammalian collagen-like triple-helical structure. These proteins mediate the internalization of GAS into human cells upon binding of Scl to the human collagen receptor integrin [60]. The GGS_124 gene encoding collagen-like protein does not contain a signal peptide or LPXTG motif, suggesting that the gene product may not be expressed on the cell surface. In contrast, GGS_124 does not harbor genes encoding proteins similar to the other collagen-like proteins (*sclA *and *sclB*) in GAS.

NAD glycohydrolase (SDEG_2029), which is located in the NADase-streptolysin O operon of the GAS genome [61], was found to be conserved in the same operon in GGS_124 (Additional file 6). This enzyme is expressed after streptolysin O is injected into host cells and accelerates cell death [61,62].

#### (x) Distribution of virulence factors among *Streptococci*

We also assessed the presence or absence of representative virulence factors among sequenced streptococcal species, including GAS (MGAS315), SESE (4047), SESZ (MGCS10565), GBS (A909), and *S. uberis *(0140J) (Additional file 8). Among 30 virulence factors, most of those located in the core genome, but not those located in streptococcal phages, are conserved in GGS_124, except for *speB*. In contrast, other *streptococci *lack genes encoding streptolysin O, NAD glycohydrolase and DRS (or SIC), suggesting the importance of these proteins in the pathogenicity of SDSE and GAS in humans, causing STSS.

### Putative virulence factors unique to SDSE

We identified 20 gene products in GGS_124 containing signal peptides and LPXTG cell wall surface anchor motifs that showed low similarity to known proteins. Using PCR, we analyzed the distributions of these putative virulence factors in 8 SDSE isolates (Table 3 arranged according to their decreasing lethality in mice). A putative T-antigen-like protein structural subunit (SDEG_0158), encoded in the FCT-6-like region in GGS_124, was detected in the 3 most virulent strains, GGS_124, GGS_168, and GGS_117. In both GGS_168 and GGS_117, the FCT-6-like region was not detected by PCR (data not shown). Interestingly, the SDEG_1601 gene encoding a functionally unknown hypothetical protein was amplified in isolates that cause STSS. However, no virulence factors associated with mouse lethality or Lancefield groups were found.

**Table 3:** Putative virulence factors found in GGS_124 and their prevalence in the SDSE isolates based on the results of PCR analyses.

*S. dysgalactiae *subsp. *equisimilis *GGS_124					Best hit strain			PCR analysis							
		
Locus tag	Product name	Length (aa)	LPXTG motif	% Identity	Strain	Product name	Reference sequence	GGS _124	168	GGS _117	170	164	GGS _118	169	163
					*Streptococcus equi*										
	fimbrial subunit				subsp. *zooepidemicus*	fimbrial subunit									
SDEG_0157	Protein	645	IPNTG	40.48	MGCS10565	protein	YP_002124169.1	Yes	x	x	x	x	x	x	x
	T-antigen-like fimbrial				*Streptococcus equi*	T-antigen-like fimbrial									
	structural subunit				subsp. *zooepidemicus*	structural subunit									
SDEG_0158	protein	315	IPKTG	49.84	MGCS10565	protein	YP_002124168.1	Yes	Yes	Yes	x	x	x	x	x
SDEG_0180	hypothetical protein	184		-	No hit	-	-	Yes	Yes	Yes	Yes	Yes	Yes	Yes	Yes
	hypothetical					hypothetical									
	membrane associated				*Streptococcus pyogenes*	membrane-associated									
SDEG_0267	protein	242		44.35	MGAS2096	Protein	YP_601272.1	Yes	Yes	Yes	Yes	Yes	Yes	Yes	Yes
	cell surface serine				*Streptococcus*										
SDEG_0574	endopeptidase	216		49.36	*Agalactiae*	CspA	AAN85092.1	Yes	Yes	Yes	Yes	Yes	Yes	Yes	Yes
					*Streptococcus equi*										
	cell wall surface				subsp. *zooepidemicus*	cell wall surface									
SDEG_0805	anchor family protein	486	LPKAG	43.73	MGCS10565	anchor family protein	YP_002123384.1	Yes	Yes	x	x	Yes	x	x	Yes
					*Streptococcus equi*										
					subsp. *zooepidemicus*	histidine triad protein									
SDEG_0918	histidine triad protein	153		51.35	MGCS10565	HtpA	YP_002123384.1	Yes	Yes	Yes	Yes	Yes	Yes	Yes	Yes
	complement inhibitor				*Streptococcus pyogenes*	complement inhibitor									
SDEG_0932	protein	226		47.92	MGAS2096	protein	YP_601343.1	Yes	x	x	x	x	Yes	x	x
	nisin resistance				*Streptococcus*	nisin resistance									
SDEG_0979	protein, putative	322		42.17	*agalactiae *2603V/R	Protein	NP_687984.1	Yes	Yes	Yes	Yes	Yes	Yes	Yes	Yes
					*Streptococcus*										
SDEG_1327	YaeC family protein	280		49.47	*agalactiae *2603V/R	YaeC family protein	NP_687791.1	Yes	Yes	Yes	Yes	Yes	Yes	Yes	Yes
	probable surface					probable surface									
	antigen negative				*Streptococcus suis*	antigen negative									
SDEG_1429	regulator	185		52.22	98HAH33	regulator Par	YP_001200806.1	Yes	x	Yes	Yes	Yes	Yes	x	Yes
					*Streptococcus pyogenes*										
SDEG_1480	hypothetical protein	299	LPVTG	33.33	MGAS6180	hypothetical protein	YP_280631.1	Yes	x	Yes	Yes	Yes	Yes	Yes	Yes
SDEG_1511	hypothetical protein	546		27.59	*Streptococcus suis*	hypothetical protein	ABQ42885.1	Yes	Yes	Yes	Yes	Yes	Yes	Yes	Yes
					*Streptococcus uberis*										
SDEG_1573	adhesion protein	661	LPKTG	38.8	UT888	adhesion protein	ABB52003.1	Yes	Yes	x	x	x	x	x	Yes
SDEG_1601	hypothetical protein	249		-	No hit	-	-	Yes	x	Yes	x	x	Yes	x	x
					*Streptococcus pyogenes*										
SDEG_1773	hypothetical protein	210	FPSTG	37.96	M1 GAS	hypothetical protein	NP_268519.1	Yes	Yes	Yes	x	x	Yes	Yes	Yes
					*Streptococcus sanguinis*										
SDEG_1969	hypothetical protein	234		32.77	SK36	hypothetical protein	YP_001035903.1	Yes	Yes	Yes	Yes	Yes	Yes	Yes	Yes
	protein F2-like				*Streptococcus equi*										
	fibronectin binding				subsp. *zooepidemicus*	fibronectin binding									
SDEG_1984	Protein	528	LPATG	40.46	ATCC 35246	protein	ABC87919.1	Yes	x	x	Yes	Yes	x	Yes	Yes
SDEG_2022	hypothetical protein	106		-	No hit	-	-	Yes	Yes	Yes	Yes	Yes	Yes	Yes	Yes
SDEG_2141	hypothetical protein	175		-	No hit	-	-	Yes	x	x	x	Yes	x	x	Yes

Genes encoding proteins, which contain predicted signal peptide based on SignalP http://www.cbs.dtu.dk/services/SignalP and the LPXTG motif based on HMMER http://bamics3.cmbi.kun.nl/jos/sortase_substrates/help.html and were not highly homologous to putative virulence factors of GGS_124. Distribution of the putative virulence factors of GGS_124 in the 16 SDSE strains was analyzed by PCR, and the results are presented as SDSE strains in order of decreasing lethality in mice. Yes: PCR analysis postive; x: PCR analysis negative.

### Relatedness of *emm *type and pathogenicity in SDSE

Particular M (or *emm*) types of GAS have been associated with certain streptococcal diseases [63]. In investigating the pathogenic traits of M4, M12, M1, and M3 GAS clinical isolates in a murine model [64], we found that murine lethality was closely associated with M type. The M1 and M3 types of GAS, which are isolated at high frequency from patients with STSS, showed higher virulence in mice than did M12 and M4. Using this mouse model, we assessed the virulence of the *emm *types of 8 group G SDSE isolates to analyze whether most frequent *emm*-type of isolates in epidemiological studies (see below) are more virulent in mice (Table 1). We found that the lethality in mice of these isolates was not associated with their isolation from patients with STSS or with the frequently isolated *emm*-type in humans such as stG10.0 and stG643.0 (see below). To determine whether the *emm *type of SDSE was associated with pathogenicity in humans, we reviewed the epidemiological data regarding the isolation frequencies of GCS and GGS in relation to their *emm *types [3,6,7,65,66] (Additional file 9). We found that *stG10.0 *was the most frequent *emm *type in Portugal and Japan, *stG643.0 *was the most frequent in western Norway, and *stG6.0 *was the most frequent in the USA. When we calculated the degree of correspondence among the isolation rates of *emm *types in each area using the Kendall tau rank correlation coefficient, we found no significant correlations among all regions (data not shown), suggesting that there is no linkage between *emm *type of SDSE and infectivity in humans or mice.

## Discussion

Comparative analysis of *Streptococcus *16S rRNA sequences had indicated that SDSE was more closely related to GBS than to GAS [67,68]. In contrast, we found that SDSE, which belongs to Lancefield groups C and G [3,65], is more closely related to GAS than to other sequenced streptococci based on genome wide and gene level comparisons

**Figure 7:**
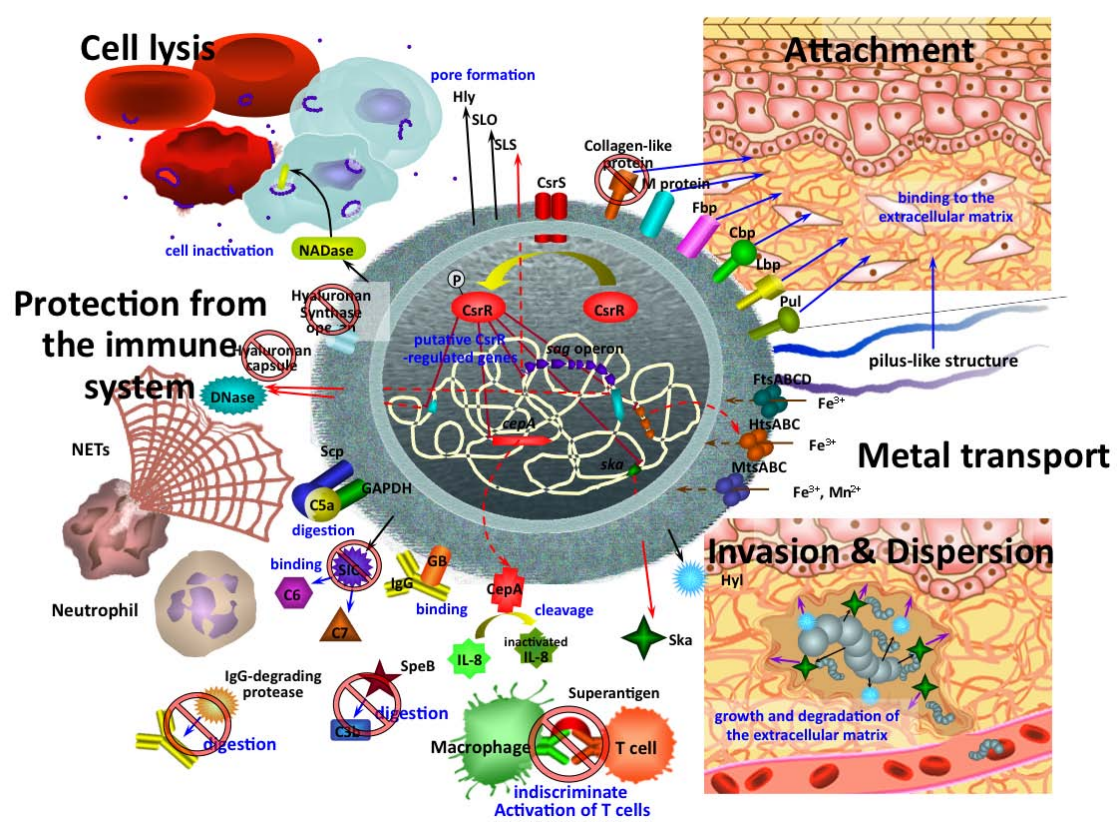
**Putative virulence factors and posited virulence function of SDSE**. Cell-surface proteins, extracellular secreted proteins, metal transporters, and the two-component regulator CsrR/CsrS, which affect the expression of approximately 10% of all genes in a GAS strain [78,79], are shown. The putative virulence factors on the cell surface of SDSE are adhesins, including M protein, fibronectin binding protein (Fbp), collagen binding protein (Cbp), laminin binding protein (Lbp), and pullulanase (Pul) [80]. Factors that protect bacteria from the host immune system are shown, including cell envelope proteinase A (CepA), which cleaves within the interleukin-8 (IL-8) C-terminal α-helix [38]; immunoglobulin G binding protein (GB) [49]; glyceraldehyde-3-phosphate dehydrogenase (GAPDH), and C5a peptidase (Scp) [46]. Putative pathogenic roles of the virulence factors of SDSE, including hyaluronidase (Hyl), streptokinase (Ska), extracellular nuclease and streptodornase (DNase), which digest neutrophil extracellular traps (NETs) released from dead neutrophils [50], and the pore-forming proteins, such as hemolysin (Hly), streptolysin S (SLS), streptolysin O (SLO), and NAD glycohydrolase (NADase), are indicated. FtsABCD and HtsABC are ferrichrome transporters and MtsABC is a metal transporter. Black arrows show protein secretion, red arrows show expression of genes regulated by CsrR, blue arrows show protein attachment to the extracellular matrix, brown arrows show metal transport from the extracellular environment into the cell, and purple arrows show degradation of extracellular matrix by secreted Hyl or Ska. The factors marked with a Stop sign, which are major virulence factors of GAS, do not function in SDSE.

SDSE is known to cause diseases very similar to those caused by GAS, such as pharyngitis, cellulitis, infective arthritis, vertebral osteomyelitis, and STSS [10-16]. This similarity may be due, at least in part, to their conservation of a large number of genes for virulence factors. Figure 7 shows a summary of putative virulence factors and proposed virulence functions in SDSE. SDSE shares most of the virulence factor genes of GAS, including streptolysin O, streptokinase, FCT-like regions, NADase, and DRS. However, GGS_124 and probably almost all other SDSEs lack SpeB, superantigens except for SpeG, and hyaluronan synthesis *via hasABC *[31]. As *spegg4*, which has about 1% of the mitogenic activity of GAS-derived SpeG, is the only gene encoding a protein homologous to superantigens, and most SDSE isolates do not harbor superantigen-like genes other than *spegg *[31,69], it is highly unlikely that superantigens play a significant role in the pathogenesis of SDSE infection in humans.

Particular M (or *emm*) types of GAS have been associated with certain streptococcal diseases [63]. We therefore analyzed the virulence in a mouse model of several SDSE strains bearing different *emm*-types, but we were unable to find a significant correlation between *emm*-type of SDSE and virulence. Further studies are needed to provide further insight into the linkage between *emm *type of SDSE and infectivity in humans.

Streptococcal phages are considered critical in horizontal gene transfer, especially in the transport of virulence factors [28,70], in *Streptococci*. Three prophage elements in GGS_124 were found to be homologous to GAS prophages in both nucleotide and amino acid sequences. The positions of prophage insertion were also conserved between GGS_124 and previously sequenced GAS prophages, suggesting that SDSE and GAS share the same phage species, and that horizontal gene transfer between SDSE and GAS has occurred. However, GGS_124 does not contain prophages that encode genes for superantigens, Sla, or MF. These phage encoded genes were also missing from all strains previously analyzed by a GAS microarray [31]. Sdn is an exception, since it was detected in 4 of these 58 SDSE strains [31]. These results showed that SDSE may have some resistance to infection by GAS phages carrying genes encoding virulence factors.

Prokaryotes possess the CRISPR/Cas system, which mediates resistance to infection by foreign DNA, such as viruses [26,27]. GGS_124 has a CRISPR/Cas system, designated CRISPR1/Cas, whereas the other SDSE isolates analyzed in this study had another CRISPR/Cas system, designated CRISPR2/Cas. We found that SDSE strains usually have a higher total number of spacers than GAS, suggesting that prophage infection of SDSE was restricted to some extent, resulting in a smaller number of virulence factors located in the prophage regions of SDSE. Similar restrictions were observed in SESZ when compared with SESE. For example, the SESE 4047 genome, which contains no CRISPR, contains genes encoding virulence factors in prophage regions. In contrast, SESZ MGCS10565 and H70, which contain 26 and 18 spacers, respectively, do not carry any prophages. Thus, the CRISPR system in streptococci sharing prophages may play a substantial role in the spread of virulence factors among species. Alternatively, these virulence factors may not benefit to SDSE during carriage or disease, such that the integration of these specific phages is not selected for.

## Conclusions

We have shown here that SDSE likely acts as a pathogen, based on its genome sequence and close relationship with GAS. As the frequency of isolation of SDSE from patients has increased, it should not be overlooked as a source of infection.

## Methods

### Bacterial strains

Three *Streptococcus dysgalactiae *subsp. *equisimilis *(SDSE) isolates, GGS_124, GGS_117, and GGS_118, were obtained from 3 patients with STSS, and 5 SDSE isolates were isolated from 5 patients with non-STSS (Table 1). All SDSE isolates were classified as Lancefield group G. *Streptococcus pyogenes *(GAS). NIH9 [71] was used as the SpeB-producing strain.

### *emm *typing

The *emm *types were classified according to a protocol for *emm *typing of the Centers for Disease Control and Prevention (CDC) http://www.cdc.gov/ncidod/biotech/strep/protocol_emm-type.htm.

### SDSE infection in mice

All animal experiments were performed according to the guidelines of the Ethics Review Committees of Animal Experiments of Tokyo Women's Medical University and the National Center for Global Health and Medicine. Virulence in mice was determined as described [64]. Briefly, LD_50 _values were determined by intraperitoneal (*i.p*.) injection of each SDSE strain into 5 6-7 week old female ddY mice.

### Genome sequencing

We obtained draft contig data of GGS_124 from a commercial service (454 Life Sciences, Branford, CT), and the gaps between the contigs were tiled by PCR after closure to validate assembly using specific primers or by primer walking and an ABI 3100 genetic analyzer (Applied Biosystems Inc., Foster City, CA). The percentage of QV40+ bases, an index of the quality of data from pyro-sequencing, was 99.87%. The genome sequence of GGS_124 has been deposited in the DDBJ database (accession no. AP010935).

### Genome annotation and bioinformatics

Transfer RNAs (tRNAs), transfer-messenger RNA (tmRNA), and rRNA sequences were predicted using ARAGORN [72]. Coding sequences (CDS) were predicted using *in silico *Molecular Cloning (In Silico Biology Inc., Yokohama City, Kanagawa, Japan) for selection of optimal start sites. Predicted genes and intergenic regions were compared using the NCBI sequence database http://blast.ncbi.nlm.nih.gov/Blast.cgi, and predicted CDS and start sites were adjusted accordingly. A genome-wide homology search was performed using the discontiguous megaBLAST algorithm http://blast.ncbi.nlm.nih.gov/Blast.cgi with a word size of 11 and rewards and penalties (2, -3) that optimize for alignments of about 85% identity. The phylogenetic tree of all sequenced *Streptococcus *species was constructed based on CVTree http://tlife.fudan.edu.cn/cvtree/[73]. Secretion signal peptides were predicted using SignalP http://www.cbs.dtu.dk/services/SignalP[74]. Sortases and cell wall sorting signals were predicted with available hidden Markov models using HMMER http://bamics3.cmbi.kun.nl/jos/sortase_substrates/help.html[75]. Clustered, regularly interspaced, short palindromic repeat (CRISPR) spacers were detected using CRISPR Finder http://crispr.u-psud.fr/[76]. The phylogenetic tree of all sequenced *Streptococcus *was constructed based on the CVTree http://tlife.fudan.edu.cn/cvtree/[73]

### Detection and sequencing of CRISPRs and CRISPR-associated protein (Cas) genes in the 8 SDSE strains

CRISPRs and Cas genes in the SDSE strains were detected by conventional PCR using ExTaq (Takara Bio Inc., Otsu, Shiga, Japan) and the primers listed in Additional file 10. Template DNA was isolated as described [64]. The PCR cycling conditions were 94°C for 2 min followed by 30 cycles of 94°C for 30 s, 54°C for 30 s, and 72°C for 4 min, and a final extension at 72°C for 6 min. The amplified CRISPR fragments were sequenced using an ABI 3100 genetic analyzer (Applied Biosystems Inc.). The CRISPR spacers were identified using CRISPR Finder http://crispr.u-psud.fr/[76]. The sequences of CRISPR and the spacers have been deposited in the DDBJ database (accession numbers are listed in Table 2).

### Determination of SpeB-like protease activity

SpeB-like protease activity was assayed as described [77], with slight modifications. Briefly, DTT was added to bacterial culture supernatants to a final concentration of 10 mM and incubated at 37°C for 30 min. An equal amount of 2 g/L azocasein was added, and incubation was continued for an Additional 30 min at 37°C. Trichloroacetic acid was added to a final concentration of 5%, and incubation was continued for 15 min at 4°C. After centrifugation, an equal amount of 5 M NaOH was added to the supernatant, and its absorbance at 450 nm was measured.

### Distribution of putative virulence factors unique to GGS_124 among the other SDSE

The distribution of putative virulence factors of GGS_124 among SDSE strains was analyzed by conventional PCR using ExTaq (Takara Bio Inc.) and the primers listed in Additional file 10. Template DNA extraction and PCR were performed as described [64].

## Authors' contributions

YS and TMA performed the molecular genetic studies, participated in sequence alignment and drafted the manuscript. KO performed the molecular genetic studies and participated in sequence alignment. SYM and KU analyzed the genome sequences. JY, YS and TMA performed animal experiments. TK drafted the manuscript. UK, JY and TK conceived of the study, participated in its design and coordination, and helped to draft the manuscript. All authors read and approved the final manuscript.

## Supplementary Material

Additional file 1 Overview and comparison of the genome sequences of GGS_124 and genus Streptococcus available in databases as of January 2010. Click here for file

Additional file 2 **Unrooted phylogenetic tree of genus *Streptococcus*, including *S. dysgalactiae *subsp *equisimilis *GGS_124**. The phylogenetic tree of all sequenced *Streptococcus *was constructed based on CVTree http://tlife.fudan.edu.cn/cvtree/[73], which constructs whole genome based phylogenetic trees without sequence alignment by using a Composition Vector (CV) approach. The genetic distances between the major nodes are shown. Click here for file

Additional file 3 **Genome rearrangement map of SDSE strain GGS_124 relative to GAS strains MGAS315 and strain SSI-1, and *S. uberis *0140J**. The genes were aligned from the predicted replication origin of each genome. The colored bars separating each genome represent similarity matches identified by *in silico *molecular cloning. BLASTP comparisons of CDS with GAS MGAS315 and SSI-1 and *S. uberis *0140J are shown as amino acid identities of ≥90% (red), 89%-80% (orange), 79%-70% (yellow), 69%-60% (green), 59%-50% (light blue), and 49%-40% (dark blue). Prophages are highlighted as green boxes. Click here for file

Additional file 4 GGS_124 genes showing higher similarity to genes from bacteria other than GAS, or no similarity to genes in the databases Click here for file

Additional file 5 Features of CRISPR found in the GGS_124 genome and phages derived from GAS containing sequences homologous to GGS_124 spacers Click here for file

Additional file 6 Putative virulence factors found in GGS_124 and their homologous genes in other *streptococcal *species Click here for file

Additional file 7 **Determination of SpeB-like protease activity in the SDSE isolates**. SDSE (GGS_124 and GGS_118) and GAS (NIH9) were cultured in BHI in the presence or absence of E-64, and the culture supernatants were analyzed for protease activity using azocasein as a substrate. The background activity of BHI is also shown. Click here for file

Additional file 8 **Distribution of the virulence factors found in SDSE among other *****streptococci*** Click here for file

Additional file 9 **Comparison of *emm*-type of GCS and GGS isolates from humans reported from 4 countries**. Shown are *emm*-specific differences of SDSE in invasive and noninvasive infections from 1998 to 2004 in Portugal [65], the *emm *types of 128 strains of SDSE collected from 11 medical institutions in Japan from September 2003 to October 2005 [7], the *emm *types of 64 GCS and GGS isolates associated with noninvasive disease in western Norway from February 2005 to March 2006 [66], and the *emm *types of 212 invasive SDSE isolates collected in Atlanta, Georgia, from July 2002 to June 2004 and in the San Francisco Bay Area of California from January 2003 to December 2004 in the USA [3]. Each stack was ordered from higher (top) to lower isolation frequency (bottom). Click here for file

Additional file 10 List of oligonucleotide primers used in this study. Click here for file
