# Supplementary material for: Complete genome sequencing and analysis of a Lancefield group G Streptococcus dysgalactiae subsp. equisimilis strain causing streptococcal toxic shock syndrome (STSS)
Source: BMC Genomics. 2011 Jan 11;12:17. doi: 10.1186/1471-2164-12-17 (PMC3027156; doi:10.1186/1471-2164-12-17)
Supplement: Additional file 1 — Overview and comparison of the genome sequences of GGS_124 and genus Streptococcus available in databases as of January 2010. [file 1471-2164-12-17-S1.PDF]

Additional file 1. Overview and comparison of the genome sequences of GGS_124 and genus *Streptococcus* available in databases as of January 2010.

									Blast results			
Species	strain	Length (nt)	G+C%	% Genome	CDS	Ave. (nt)	rRNAs	tRNA	Max score	Total score	Query coverage	Acc. No.
S. dysgalactiae subsp. *equisimilis*	GGS_124	2,106,340	39.6	86.9	2,095	873	5	57	3.80E+06	5.04E+06	100%	AP010935.1
S. pyogenes	MGAS8232	1,895,017	38.6	84.8	1,839	875	6	67	4.55E+04	2.38E+06	72%	AE009949.1
	MGAS10394	1,899,877	38.7	86.8	1,886	878	6	65	3.55E+04	2.29E+06	71%	CP000003.1
	MGAS10750	1,937,111	38.3	86.9	1,979	855	6	63	4.57E+04	2.23E+06	74%	CP000262.1
	Manfredo	1,841,271	38.6	83.5	1,745	883	6	66	3.21E+04	2.19E+06	72%	AM295007.1
	MGAS10270	1,928,252	38.4	86.9	1,986	848	6	63	4.55E+04	2.10E+06	73%	CP000260.1
	MGAS315	1,900,521	38.6	85.5	1,865	874	6	68	4.56E+04	2.10E+06	72%	AE014074.1
	MGAS5005	1,838,554	38.5	86.4	1,865	854	6	65	4.57E+04	2.09E+06	72%	CP000017.1
	MGAS9429	1,836,467	38.5	87.2	1,877	858	6	67	4.56E+04	2.08E+06	70%	CP000259.1
	MGAS2096	1,860,355	38.7	86.8	1,898	856	6	63	4.56E+04	2.07E+06	70%	CP000261.1
	SF370	1,852,441	38.5	83.6	1,697	914	6	61	4.57E+04	2.06E+06	72%	AE004092.1
	SSI-1	1,894,275	38.6	84.7	1,861	864	5	57	4.56E+04	2.05E+06	72%	BA000034.2
	MGAS6180	1,897,573	38.4	86.6	1,894	871	6	65	4.57E+04	2.05E+06	71%	CP000056.1
	NZ131	1,815,785	38.6	84.6	1,700	907	6	66	4.56E+04	2.05E+06	70%	CP000829.1
S. equi subsp. *equi*	4047	2,253,973	41.3	85.1	2,137	931	6	66	1.38E+04	1.33E+06	64%	FM204883.1
S. equi subsp. *zooepidemicus*	MGCS10565	2,024,171	41.8	82.7	1,893	878	5	57	1.16E+04	1.19E+06	65%	CP001129.1
	H70	2,149,868	41.5	82.4	1,960	951	5	57	1.08E+04	1.15E+06	64%	FM204884.1
S. agalactiae	A909	2,127,839	35.6	86	1,996	918	7	80	1.34E+04	1.09E+06	57%	CP000114.1
	2603 V/R	2,160,267	35.7	86.3	2,124	880	7	80	1.27E+04	1.08E+06	56%	AE009948.1
	NEM316	2,211,485	35.6	87.5	2,094	926	7	80	1.34E+04	1.05E+06	55%	AL732656.1
S. uberis	0140J	1,852,352	36.6	86.9	1,760	916	5	59	1.31E+04	9.92E+05	58%	AM946015.1
S. thermophilus	CNRZ1066	1,796,226	39.1	83.5	1,915	787	6	67	9634	8.11E+05	42%	CP000024.1
	LMD-9	1,856,368	39.1	76.8	1,710	835	6	68	9638	8.04E+05	42%	CP000023.1
	LMG18311	1,796,846	39.1	83.6	1,889	799	6	64	9604	7.74E+05	41%	CP000419.1
S. mutans	NN2025	2,013,587	36.9	85.5	1,904	912	5	65	6269	7.71E+05	47%	AP010655.1
	UA159	2,030,921	36.8	85.6	1,960	890	5	64	4695	7.52E+05	47%	AE014133.1
S. pneumoniae	70585	2,184,682	39.7	84.3	2,202	838	4	58	9465	6.53E+05	42%	CP000918.1
	ATCC 700669	2,221,315	39.5	82.4	1,990	921	4	58	9449	6.50E+05	41%	FM211187.1
	CGSP14	2,209,198	39.5	86.2	2,206	869	4	58	9436	6.49E+05	41%	CP001033.1
	Hungary19-A6	2,245,615	39.6	82.6	2,155	863	4	55	9449	6.45E+05	41%	CP000936.1
	TIGR4	2,160,842	39.7	83.2	2,105	857	4	58	9449	6.41E+05	41%	AE005672.3
	JJA	2,120,234	39.7	84	2,123	841	4	58	9443	6.40E+05	41%	CP000919.1
	P1031	2,111,882	39.8	83	2,073	849	4	58	9441	6.38E+05	41%	CP000920.1
	D39	2,046,115	39.7	83.5	1,914	894	4	58	9458	6.37E+05	41%	CP000410.1
	R6	2,038,615	39.7	86.3	2,043	867	4	58	9458	6.37E+05	41%	AE007317.1
	Taiwan19F-14	2,112,148	39.8	82.7	2,044	857	4	58	9445	6.37E+05	41%	CP000921.1
	G54	2,078,953	39.6	85.7	2,115	845	4	57	9452	6.35E+05	41%	CP001015.1
S. gordonii	Challis substr. (	2,196,662	40.5	87.7	2,051	942	4	59	8262	6.23E+05	42%	CP000725.1
S. suis	BM407	2,146,229	41.1	83.7	1,932	932	4	52	6506	6.26E+05	41%	FM252032.1
	P1/7	2,007,491	41.3	84.7	1,824	935	4	56	6506	6.21E+05	41%	AM946016.1
	SC84	2,095,898	41.1	84.6	1,898	937	4	56	6506	6.21E+05	41%	FM252031.1
	98HAH33	2,095,698	41.1	86.4	2,185	841	4	56	6412	6.18E+05	41%	CP000408.1
	05ZYH33	2,096,309	41.1	86.3	2,186	841	4	56	6478	6.17E+05	41%	CP000407.1
S. sanguinis	SK36	2,388,435	43.4	88.3	2,270	933	4	62	9310	6.10E+05	42%	CP000387.1

The entire genome sequence of GGS_124 was used for comparison with the discontiguous megablast algorithm (http://blast.ncbi.nlm.nih.gov/Blast.cgi). Max score: The highest alignment score of a set of aligned segments from the same subject (database) sequence. Total score: The sum of alignment scores of all segments from the same subject sequence. Query coverage: The percentage query length included in the aligned segments.
