# Supplementary figures and images for: Complete genome sequencing and analysis of a Lancefield group G Streptococcus dysgalactiae subsp. equisimilis strain causing streptococcal toxic shock syndrome (STSS)
Source: BMC Genomics. 2011 Jan 11;12:17. doi: 10.1186/1471-2164-12-17 (PMC3027156; doi:10.1186/1471-2164-12-17)

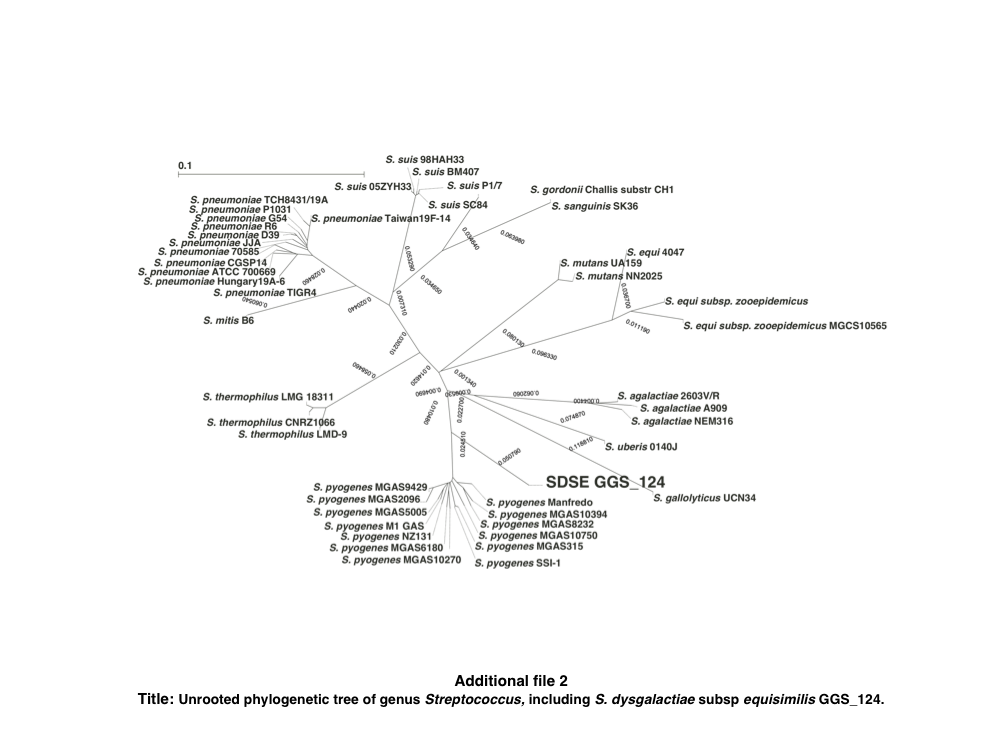

Supplement: Additional file 2 — Unrooted phylogenetic tree of genus Streptococcus, including S. dysgalactiae subsp equisimilis GGS_124. The phylogenetic tree of all sequenced Streptococcus was constructed based on CVTree http://tlife.fudan.edu.cn/cvtree/[73], which constructs whole genome based phylogenetic trees without sequence alignment by using a Composition Vector (CV) approach. The genetic distances between the major nodes are shown. [file 1471-2164-12-17-S2.TIFF]

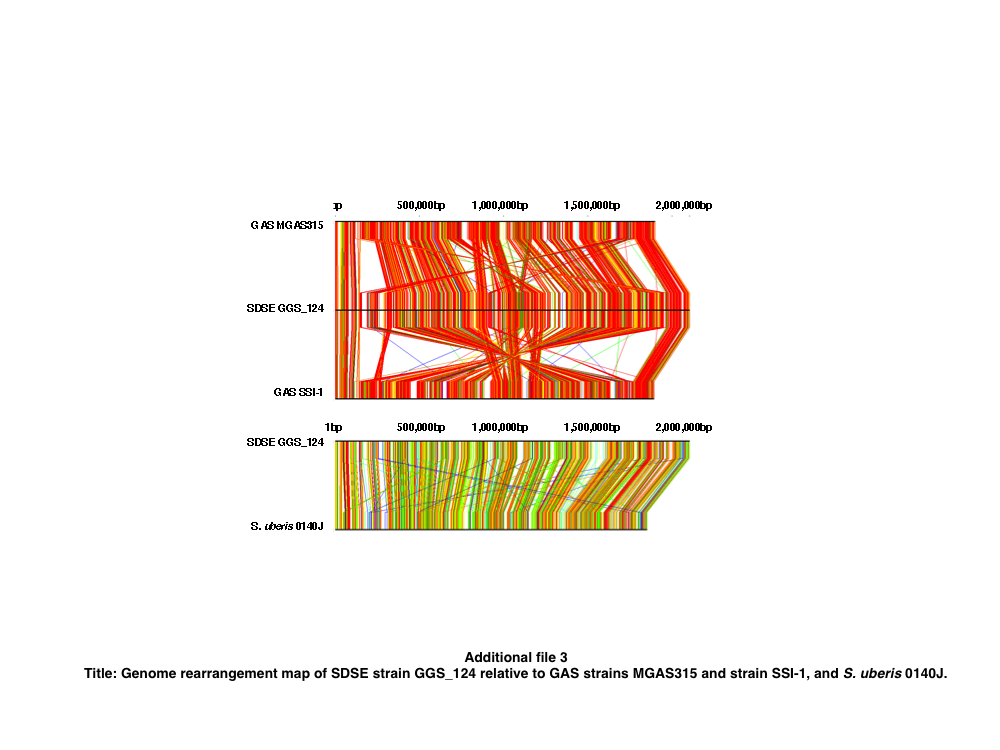

Supplement: Additional file 3 — Genome rearrangement map of SDSE strain GGS_124 relative to GAS strains MGAS315 and strain SSI-1, and S. uberis 0140J. The genes were aligned from the predicted replication origin of each genome. The colored bars separating each genome represent similarity matches identified by in silico molecular cloning. BLASTP comparisons of CDS with GAS MGAS315 and SSI-1 and S. uberis 0140J are shown as amino acid identities of ≥90% (red), 89%-80% (orange), 79%-70% (yellow), 69%-60% (green), 59%-50% (light blue), and 49%-40% (dark blue). Prophages are highlighted as green boxes. [file 1471-2164-12-17-S3.TIFF]

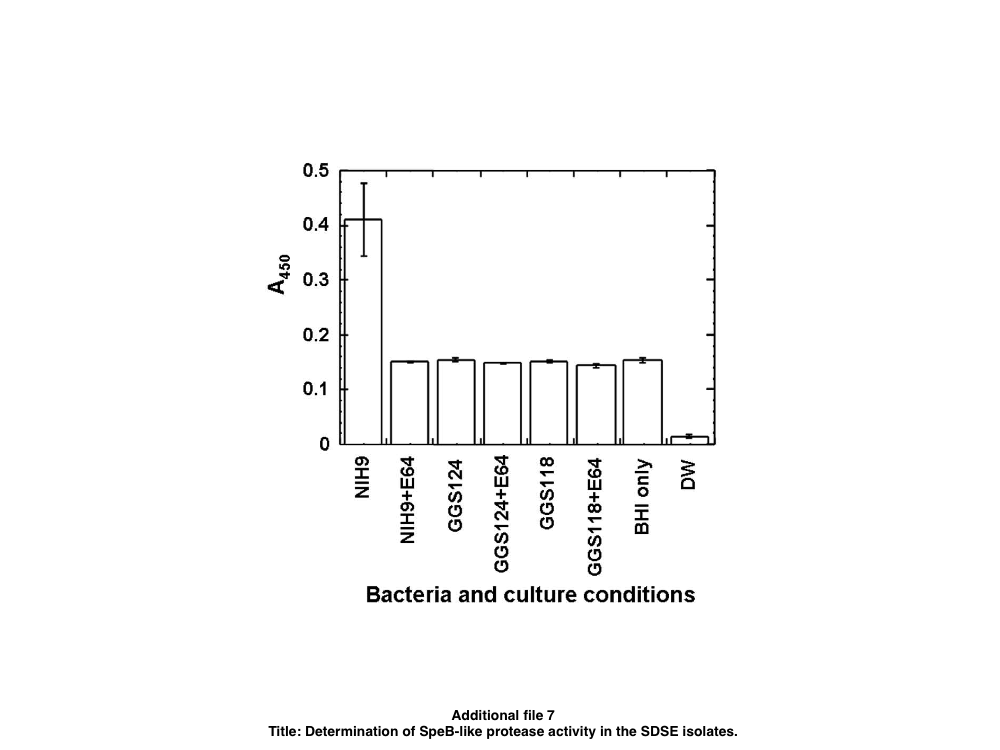

Supplement: Additional file 7 — Determination of SpeB-like protease activity in the SDSE isolates. SDSE (GGS_124 and GGS_118) and GAS (NIH9) were cultured in BHI in the presence or absence of E-64, and the culture supernatants were analyzed for protease activity using azocasein as a substrate. The background activity of BHI is also shown. [file 1471-2164-12-17-S7.TIFF]

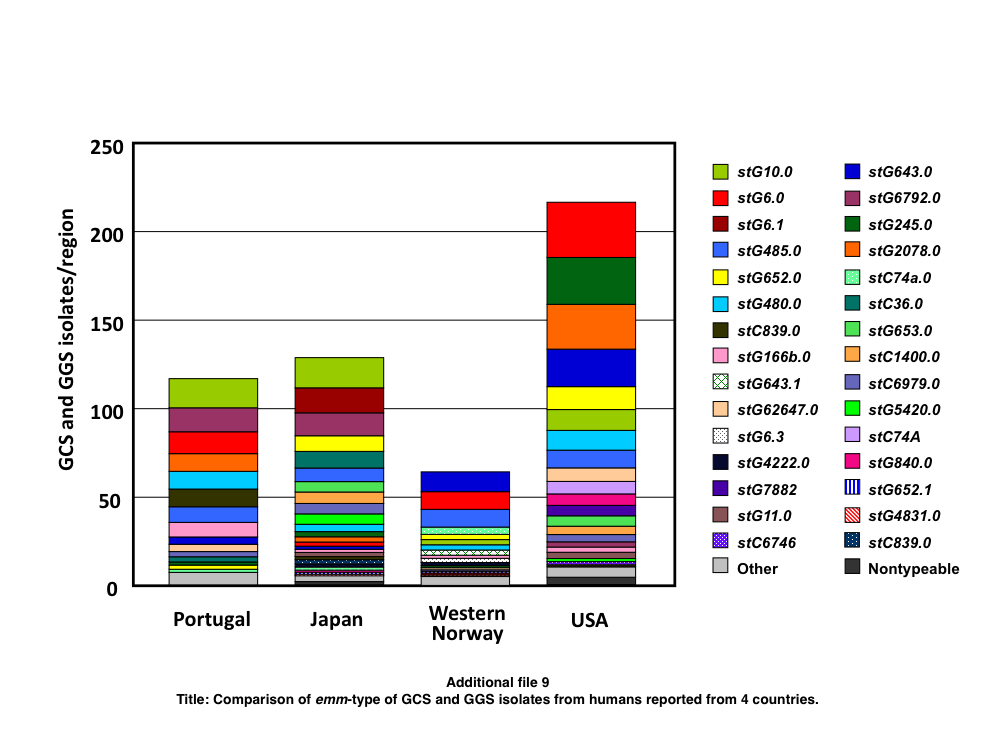

Supplement: Additional file 9 — Comparison of emm-type of GCS and GGS isolates from humans reported from 4 countries. Shown are emm-specific differences of SDSE in invasive and noninvasive infections from 1998 to 2004 in Portugal [65], the emm types of 128 strains of SDSE collected from 11 medical institutions in Japan from September 2003 to October 2005 [7], the emm types of 64 GCS and GGS isolates associated with noninvasive disease in western Norway from February 2005 to March 2006 [66], and the emm types of 212 invasive SDSE isolates collected in Atlanta, Georgia, from July 2002 to June 2004 and in the San Francisco Bay Area of California from January 2003 to December 2004 in the USA [3]. Each stack was ordered from higher (top) to lower isolation frequency (bottom). [file 1471-2164-12-17-S9.TIFF]
