# Supplementary material for: Complete genome sequencing and analysis of a Lancefield group G Streptococcus dysgalactiae subsp. equisimilis strain causing streptococcal toxic shock syndrome (STSS)
Source: BMC Genomics. 2011 Jan 11;12:17. doi: 10.1186/1471-2164-12-17 (PMC3027156; doi:10.1186/1471-2164-12-17)
Supplement: Additional file 4 — GGS_124 genes showing higher similarity to genes from bacteria other than GAS, or no similarity to genes in the databases [file 1471-2164-12-17-S4.PDF]

Additional file 4. GGS_124 genes showing higher similarity to genes from bacteria other than GAS, or no similarity to genes in the databases

Genes showing similarity to Streptococcal genes other than those of GAS		
SDEG_0004	similar to hypothetical protein gbs0005	Streptococcus agalactiae NEM316
SDEG_0063	catalyzes the formation of L-threonine from O-phospho-L-homoserine	Streptococcus agalactiae 2603V/R
SDEG_0088	similar to ribosomal protein	Streptococcus agalactiae CJB111
SDEG_0118	catalyzes the formation of dimethylmenaquinone from1,4-dihydroxy-2-naphthoate and octaprenyl diphosphate	Streptococcus agalactiae 2603V/R
SDEG_0119	similar to hypothetical protein gbs1788	Streptococcus agalactiae NEM316
SDEG_0120	similar to cytochrome d ubiquinol oxidase subunit II	Streptococcus agalactiae A909
SDEG_0121	similar to cytochrome d ubiquinol oxidase subunit II	Streptococcus agalactiae 2603V/R
SDEG_0122	similar to hypothetical protein gbs1785	Streptococcus agalactiae NEM316
SDEG_0123	similar to hypothetical protein gbs1784	Streptococcus agalactiae NEM316
SDEG_0124	similar to hypothetical protein gbs1783	Streptococcus agalactiae NEM316
SDEG_0185	similar to hypothetical protein SAG0685	Streptococcus agalactiae 2603V/R
SDEG_0198	similar to streptomycin resistance protein	Streptococcus agalactiae 2603V/R
SDEG_0202	similar to hypothetical protein gbs1981	Streptococcus agalactiae NEM316
SDEG_0205	similar to ISSdy1 transposase OrfB	Streptococcus agalactiae 2603V/R
SDEG_0206	similar to ISSdy1 transposase OrfA	Streptococcus agalactiae 515
SDEG_0224	similar to hypothetical protein SAG1944	Streptococcus agalactiae 2603V/R
SDEG_0226	similar to hypothetical protein gbs0573	Streptococcus agalactiae NEM316
SDEG_0227	similar to inosine-uridine preferring nucleoside hydrolase	Streptococcus agalactiae 2603V/R
SDEG_0279	similar to phage integrase family site specific recombinase	Streptococcus agalactiae A909
SDEG_0280	similar to transcriptional regulator Cro/CI family	Streptococcus agalactiae CJB111
SDEG_0281	similar to hypothetical protein SAG0219	Streptococcus agalactiae 2603V/R
SDEG_0282	similar to hypothetical protein SAG0220	Streptococcus agalactiae 2603V/R
SDEG_0283	similar to hypothetical protein SAG0221	Streptococcus agalactiae 2603V/R
SDEG_0284	similar to hypothetical protein SAG0222	Streptococcus agalactiae 2603V/R
SDEG_0285	similar to conserved hypothetical protein	Streptococcus agalactiae CJB111
SDEG_0287	similar to hypothetical protein SAG0225	Streptococcus agalactiae 2603V/R
SDEG_0288	similar to recombination protein	Streptococcus agalactiae 2603V/R
SDEG_0289	similar to hypothetical protein SAG0227	Streptococcus agalactiae 2603V/R
SDEG_0297	similar to the putative ATP-dependent DNA helicase recG	Streptococcus agalactiae 515
SDEG_0305	similar to the putative galactitol-specific IIA component of the PTS system	Streptococcus agalactiae A909
SDEG_0306	similar to the putative galactitol-specific IIC component of the PTS system	Streptococcus agalactiae A909
SDEG_0307	similar to the putative galactitol-specific IIB component of the PTS system	Streptococcus agalactiae A909
SDEG_0379	similar to biotin synthetase	Streptococcus agalactiae 2603V/R
SDEG_0440	similar to hypothetical protein gbs2095	Streptococcus agalactiae NEM316
SDEG_0460	similar to hypothetical protein SAG1540	Streptococcus agalactiae 2603V/R
SDEG_0461	similar to glyoxalase family protein	Streptococcus agalactiae 2603V/R
SDEG_0462	similar to sugar-binding transcriptional regulator	Streptococcus agalactiae 2603V/R
SDEG_0463	similar to HAD superfamily hydrolase	Streptococcus agalactiae 2603V/R
SDEG_0477	similar to branched-chain amino acid transportsystem II carrier protein	Streptococcus agalactiae 2603V/R
SDEG_0481	similar to conserved hypothetical protein	Streptococcus agalactiae H36B
SDEG_0526	similar to CAAX amino terminal protease family protein	Streptococcus agalactiae NEM316
SDEG_0528	similar to hypothetical protein SAG1491	Streptococcus agalactiae 2603V/R
SDEG_0532	similar to ABC transporter ATP binding protein	Streptococcus agalactiae 2603V/R
SDEG_0533	similar to hypothetical protein SAG1486	Streptococcus agalactiae 2603V/R
SDEG_0546	converts 1,4-alpha-D-glucans to maltodextrin	Streptococcus agalactiae 2603V/R
SDEG_0566	similar to hypothetical protein gbs0689	Streptococcus agalactiae NEM316
SDEG_0567	similar to amino acid ABC transporter, amino acid-binding protein	Streptococcus agalactiae 2603V/R
SDEG_0574	similar to CspA	Streptococcus agalactiae
SDEG_0575	similar to reticulocyte binding protein	Streptococcus agalactiae 515
SDEG_0608	similar to hypothetical protein SAG1433	Streptococcus agalactiae 2603V/R
SDEG_0609	similar to ammonium transporter (Amt) family protein	Streptococcus agalactiae A909
SDEG_0626	similar to hypothetical protein SAG1552	Streptococcus agalactiae 2603V/R
SDEG_0627	similar to hypothetical protein gbs1605	Streptococcus agalactiae NEM316
SDEG_0628	similar to glycosyl transferase group 2 family protein	Streptococcus agalactiae 2603V/R
SDEG_0633	similar to ISSdy1 transposase OrfB	Streptococcus agalactiae 2603V/R
SDEG_0634	similar to hypothetical protein gbs1310	Streptococcus agalactiae NEM316
SDEG_0656	similar to hypothetical protein SAG0515	Streptococcus agalactiae 2603V/R
SDEG_0660	similar to fructose-16-bisphosphatase	Streptococcus agalactiae 515
SDEG_0785	similar to ribosomal protein L21	Streptococcus agalactiae CJB111
SDEG_0816	similar to hypothetical protein SAG1344	Streptococcus agalactiae 2603V/R
SDEG_0921	similar to ISSdy1, transposase OrfB	Streptococcus agalactiae 2603V/R
SDEG_0922	similar to ISSag4, transposase OrfA	Streptococcus agalactiae A909
SDEG_0948	similar to Na+/H+ antiporter	Streptococcus agalactiae A909
SDEG_0965	similar to DNA-binding response regulator	Streptococcus agalactiae 2603V/R
SDEG_0966	similar to sensor histidine kinase	Streptococcus agalactiae 2603V/R
SDEG_0979	similar to the putative nisin-resistance protein	Streptococcus agalactiae 2603V/R
SDEG_1029	similar to ISSdy1, transposase OrfA	Streptococcus agalactiae 2603V/R
SDEG_1030	similar to putative transposase	Streptococcus agalactiae
SDEG_1224	similar to hypothetical protein SAM 0929	Streptococcus agalactiae CJB111
SDEG_1258	similar to hypothetical protein gbs1118	Streptococcus agalactiae NEM316
SDEG_1260	similar to hypothetical protein SAG0634	Streptococcus agalactiae 2603V/R
SDEG_1261	similar to ISSag4 transposase orfA	Streptococcus agalactiae A909
SDEG_1262	similar to hypothetical protein gbs1153	Streptococcus agalactiae NEM316
SDEG_1323	similar to hypothetical protein SAG0689	Streptococcus agalactiae 2603V/R
SDEG_1324	similar to ABC transporter ATP-binding protein	Streptococcus agalactiae 2603V/R
SDEG_1327	similar to YaeC family protein	Streptococcus agalactiae 2603V/R
SDEG_1328	similar to ISSdy1, transposase OrfA	Streptococcus agalactiae 515
SDEG_1329	similar to ISSdy1, transposase OrfB	Streptococcus agalactiae 2603V/R
SDEG_1402	similar to ISSdy1, transposase OrfB	Streptococcus agalactiae 2603V/R
SDEG_1403	similar to ISSag4, transposase orfA	Streptococcus agalactiae A909
SDEG_1431	similar to hypothetical protein SAK 0933	Streptococcus agalactiae A909
SDEG_1440	probable ORF of C-terminal portion of major facilitator family protein	Streptococcus agalactiae 2603V/R
SDEG_1441	probable ORF of N-terminal portion of major facilitator family protein	Streptococcus agalactiae 2603V/R
SDEG_1456	similar to YaeC family protein	Streptococcus agalactiae 2603V/R
SDEG_1457	similar to ABC transporter permease protein	Streptococcus agalactiae 2603V /R
SDEG_1458	similar to ABC transporter ATP-binding protein	Streptococcus agalactiae 2603V/R
SDEG_1523	similar to hypothetical protein gbs0508	Streptococcus agalactiae NEM316
SDEG_1525	similar to ABC transporter, ATP-binding/permease protein	Streptococcus agalactiae A909
SDEG_1526	similar to VanZF domain protein	Streptococcus agalactiae COH1
SDEG_1775	similar to transposase IS256 family	Streptococcus agalactiae 2603V/R
SDEG_1784	similar to ISSdy1 transposase OrfB	Streptococcus agalactiae 2603V/R
SDEG_1785	similar to hypothetical protein gbs1310	Streptococcus agalactiae NEM316
SDEG_1840	catalyzes the formation of phosphoenolpyruvate from pyruvate	Streptococcus agalactiae NEM316

SDEG_1841	similar to hypothetical protein SAG1671	Streptococcus agalactiae 2603V/R
SDEG_1842	similar to CBS domain protein	Streptococcus agalactiae 2603V/R
SDEG_1853	similar to oxidoreductase aldo/keto reductase family	Streptococcus agalactiae 2603V/R
SDEG_1983	similar to 5-formyltetrahydrofolate cyclo-ligase family protein	Streptococcus agalactiae 2603V/R
SDEG_2019	similar to ISSag4 transposase orfA	Streptococcus agalactiae A909
SDEG_2020	similar to ISSdy1 transposase OrfB	Streptococcus agalactiae 2603V/R
SDEG_2021	similar to ISSdy1 transposase OrfB	Streptococcus agalactiae 2603V/R
SDEG_2150	similar to hypothetical protein SAG2143	Streptococcus agalactiae 2603V/R
SDEG_1020	plasminogen activator; similar to streptokinase precursor	Streptococcus dysgalactiae subsp. equisimilis
SDEG_1989	similar to hypothetical protein	Streptococcus dysgalactiae subsp. equisimilis
SDEG_0008	similar to polysaccharide biosynthesis family protein	Streptococcus equi subsp. zooepidemicus MGCS10565
SDEG_0117	similar to putative nudix hydrolase	Streptococcus equi subsp. zooepidemicus MGCS10565
SDEG_0144	similar to membrane-bound protease CAAX family	Streptococcus equi subsp. zooepidemicus MGCS10565
SDEG_0157	similar to fimbrial subunit protein FszF	Streptococcus equi subsp. zooepidemicus MGCS10565
SDEG_0191	similar to phosphoglycerate mutase family protein	Streptococcus equi subsp. zooepidemicus MGCS10565
SDEG_0195	similar to putative transcriptional regulator	Streptococcus equi subsp. zooepidemicus MGCS10565
SDEG_0199	similar to phosphoglycerate mutase family protein	Streptococcus equi subsp. zooepidemicus MGCS10565
SDEG_0221	similar to HTH-type transcriptional regulator MalR-like	Streptococcus equi subsp. zooepidemicus MGCS10565
SDEG_0246	similar to hypothetical protein ProS	Streptococcus equi subsp. zooepidemicus MGCS10565
SDEG_0319	catalyzes the formation of 4-phospho-L-aspartate from L-aspartate and ATP; lysine and threonine sensitive	Streptococcus equi subsp. zooepidemicus MGCS10565
SDEG_0325	similar to alpha-amylase precursor AmyE	Streptococcus equi subsp. zooepidemicus MGCS10565
SDEG_0326	similar to alpha-amylase precursor AmyE	Streptococcus equi subsp. zooepidemicus MGCS10565
SDEG_0377	similar to internalin A-like histidine triadlipoprotein	Streptococcus equi subsp. zooepidemicus MGCS10565
SDEG_0457	similar to putative acetyltransferase	Streptococcus equi
SDEG_0624	similar to hypothetical protein Sez 1324	Streptococcus equi subsp. zooepidemicus MGCS10565
SDEG_0680	similar to PTS systemmannose/fructose IIB component	Streptococcus equi subsp. zooepidemicus MGCS10565
SDEG_0698	similar to cell division protein FtsW	Streptococcus equi subsp. zooepidemicus MGCS10565
SDEG_0719	catalyzes the transfer of a segment of a1,4-alpha-D-glucan chain to a primary hydroxy group in a similar glucan chain	Streptococcus equi subsp. zooepidemicus MGCS10565
SDEG_0720	catalyzes the formation of ADP-glucose and diphosphate from ATP and alpha-D-glucose 1-phosphate	Streptococcus equi subsp. zooepidemicus MGCS10565
SDEG_0721	similar to glucose-1-phosphate adenylyltransferase GlgC	Streptococcus equi subsp. zooepidemicus MGCS10565
SDEG_0722	catalyzes the formation of alpha-1,4-glucan chains from ADP-glucose	Streptococcus equi subsp. zooepidemicus MGCS10565
SDEG_0761	lytic transglycosylase, dTDP-4-rhamnose reductase; similar to glycosyltransferase lytic transglycosylased TDP-4-rhamnosereductase	Streptococcus equi subsp. zooepidemicus MGCS10565
SDEG_0763	similar to UDP-glucose 4-epimerase GalE	Streptococcus equi subsp. zooepidemicus MGCS10565
SDEG_0792	similar to membrane-associated alkaline phosphatase	Streptococcus equi subsp. zooepidemicus MGCS10565
SDEG_0805	similar to cell wall surface anchor family protein	Streptococcus equi subsp. zooepidemicus MGCS10565
SDEG_0821	similar to ABC transporter ATP-binding/permease protein	Streptococcus equi subsp. zooepidemicus MGCS10565
SDEG_0822	similar to ABC transporter ATP-binding/permease protein	Streptococcus equi subsp. zooepidemicus MGCS10565
SDEG_0823	converts 2-oxoglutarate to glutamate	Streptococcus equi subsp. zooepidemicus MGCS10565
SDEG_0832	similar to ABC transporter ATP-binding /permeaseprotein	Streptococcus equi subsp. zooepidemicus MGCS10565
SDEG_0840	similar to fibronectin-binding protein	Streptococcus equi subsp. zooepidemicus
SDEG_0919	similar to extracellular protein	Streptococcus equi subsp. zooepidemicus MGCS10565
SDEG_0975	similar to TCS senso kinase BceS	Streptococcus equi subsp. zooepidemicus MGCS10565
SDEG_0976	similar to TCS response regulator BceR	Streptococcus equi subsp. zooepidemicus MGCS10565
SDEG_0977	similar to bacitracin export permease protein BceB	Streptococcus equi subsp. zooepidemicus MGCS10565
SDEG_0995	similar to putative ammonia monooxygenase	Streptococcus equi subsp. zooepidemicus MGCS10565
SDEG_1025	catalyzes the formation of 4-aspartyl phosphate from aspartate 4-semialdehyde	Streptococcus equi subsp. zooepidemicus MGCS10565
SDEG_1027	similar to C5a peptidase precursor ScpZ	Streptococcus equi subsp. zooepidemicus MGCS10565
SDEG_1050	Catalyzes the reversible phosphorolysis of pyrimidines in the nucleotide synthesis salvage pathway	Streptococcus equi subsp. zooepidemicus MGCS10565
SDEG_1183	similar to acetyltransferase GNAT family	Streptococcus equi subsp. zooepidemicus MGCS10565
SDEG_1223	catalyzes the formation of nucleoside triphosphate from ATP and nucleoside diphosphate	Streptococcus equi subsp. zooepidemicus MGCS10565
SDEG_1244	similar to hypothetical membrane associated protein	Streptococcus equi subsp. zooepidemicus MGCS10565
SDEG_1317	similar to aminopeptidase PepS	Streptococcus equisubsp. zooepidemicus MGCS10565
SDEG_1331	similar to ABC transporter ATP-binding protein	Streptococcus equi subsp.
SDEG_1435	similar to hydrolase haloacid dehalogenase-like family	Streptococcus equi subsp. zooepidemicus MGCS10565
SDEG_1436	similar to acetyltransferase GNAT family	Streptococcus equi subsp. zooepidemicus MGCS10565
SDEG_1465	similar to cell division protein FtsI	Streptococcus equi subsp. zooepidemicus MGCS10565
SDEG_1474	similar to positive transcriptional regulator MutRfamily	Streptococcus equi subsp. zooepidemicus MGCS10565
SDEG_1487	similar to sugar kinase	Streptococcus equi subsp. zooepidemicus MGCS10565
SDEG_1519	similar to TCS response regulator	Streptococcus equi subsp. zooepidemicus MGCS10565
SDEG_1520	similar to TCS sensor histidine kinase	Streptococcus equi subsp. zooepidemicus MGCS10565
SDEG_1521	similar to ABC transporter permease protein	Streptococcus equi subsp. zooepidemicus MGCS10565
SDEG_1522	similar to multi drug transpot ATP-binding protein	Streptococcus equi subsp.
SDEG_1533	similar to D-ribose-binding protein precursor RbsB	Streptococcus equi subsp.
SDEG_1535	cytoplasmic mutarotase that catalyzes the conversion between the beta-pyran and beta-furan forms of D-ribose	Streptococcus equi subsp. zooepidemicus MGCS10565
SDEG_1536	similar to ribokinase RbsK	Streptococcus equisubsp. zooepidemicus MGCS10565
SDEG_1593	similar to phosphinothricin N-acetyltransferase	Streptococcus equi subsp. zooepidemicus MGCS10565
SDEG_1668	similar to hypothetical protein Sez 1543	Streptococcus equi subsp. zooepidemicus MGCS10565
SDEG_1720	similar to amino-acid ABC transporter; extracellular-binding protein precursor	Streptococcus equi subsp. zooepidemicus MGCS10565
SDEG_1782	similar to transcriptional regulator AraC family	Streptococcus equi subsp. zooepidemicus MGCS10565
SDEG_1810	similar to hypothetical protein Sez 0419	Streptococcus equi subsp. zooepidemicus MGCS10565
SDEG_1835	similar to drug/metabolite transporter (DMT) superfamily protein	Streptococcus equi subsp. zooepidemicus MGCS10565
SDEG_1888	Involved in disulfide oxidoreductase activity and electron transport	Streptococcus equi subsp. zooepidemicus MGCS10565
SDEG_1928	similar to oligopeptide transport system permeaseprotein OppC	Streptococcus equi subsp. zooepidemicus MGCS10565
SDEG_1929	similar to oligopeptide transport system permease protein OppB	Streptococcus equi subsp. zooepidemicus MGCS10565
SDEG_1930	similar to oligopeptide-binding protein OppAprecursor	Streptococcus equi subsp. zooepidemicus MGCS10565
SDEG_1984	similar to fibronectin-binding protein	Streptococcus equi subsp. zooepidemicus
SDEG_2039	similar to MutT/Nudix family protein	Streptococcus equi subsp. zooepidemicus MGCS10565
SDEG_2095	similar to ABC transporter ATP-binding protein	Streptococcus equi subsp. zooepidemicus MGCS10565
SDEG_2154	similar to phosphoglycolate phosphatase	Streptococcus equi subsp. zooepidemicus MGCS10565
SDEG_2086	similar to glyoxalase family protein superfamily	Streptococcus gordonii str. Challis substr. CH1
SDEG_0054	similar to hypothetical protein SGO 2086	Streptococcus gordonii str. Challis substr. CH1
SDEG_1475	similar to transporter probable SP1116	Streptococcus gordonii str. Challis substr. CH1
SDEG_0183	similar to YbaK / prolyl-tRNA synthetases associated domain	Streptococcus gordonii str. Challis substr. CH1
SDEG_1395	similar to a putative haloacid dehalogenase-like hydrolase	Streptococcus gordonii str. Challis substr.CH1
SDEG_1826	similar to 4-methyl-5(beta-hydroxyethyl)-thiazolemonophosphate synthesis protein	Streptococcus gordoniistr. Challis substr. CH1
SDEG_0456	catalyzes the formation of 3-phosphonooxypyruvateand glutamate from O-phospho-L-serine and 2-oxoglutarate; required both in the major phosphorylated pathway of serine biosynthesis and in the biosynthesis of pyridoxine	Streptococcus infantarius subsp. infantarius ATCC BAA-102
SDEG_1219	similar to hypothetical protein STRINF 01005	Streptococcus infantarius subsp. infantarius ATCCBAA-102

SDEG_1388	similar to hypothetical protein STRINF 01174	Streptococcus infantarius subsp. infantarius ATCCBAA-102
SDEG_1846	similar to hypothetical protein STRINF 01219	Streptococcus infantarius subsp. infantarius ATCCBAA-102
SDEG_1844	similar to hypothetical protein STRINF 01221	Streptococcus infantarius subsp. infantarius ATCCBAA-102
SDEG_1843	similar to hypothetical protein STRINF 01222	Streptococcus infantarius subsp. infantarius ATCCBAA-102
SDEG_1220	similar to hypothetical protein STRINF 01223	Streptococcus infantarius subsp. infantarius ATCCBAA-102
SDEG_1273	similar to hypothetical protein STRINF 01350	Streptococcus infantarius subsp. infantarius ATCCBAA-102
SDEG_0875	similar to hypothetical protein STRINF 01402	Streptococcus infantarius subsp. infantarius ATCCBAA-102
SDEG_1413	similar to hypothetical protein STRINF 01402	Streptococcus infantarius subsp. infantarius ATCCBAA-102
SDEG_1415	similar to hypothetical protein STRINF 01404	Streptococcus infantarius subsp. infantarius ATCCBAA-102
SDEG_1217	similar to hypothetical protein STRINF 01596	Streptococcus infantarius subsp. infantarius ATCCBAA-102
SDEG_0448	similar to hypothetical protein STRINF 01997	Streptococcus infantarius subsp. infantarius ATCCBAA-102
SDEG_0465	similar to hemolysis inducing protein	Streptococcus mutans UA159
SDEG_1410	similar to hypothetical protein SMU.1108c	Streptococcus mutans UA159
SDEG_0669	similar to hypothetical protein SMU.1307c	Streptococcus mutans UA159
SDEG_0252	similar to hypothetical protein SMU.145	Streptococcus mutans UA159
SDEG_1491	similar to hypothetical protein SMU.571	Streptococcus mutans UA159
SDEG_0441	similar to hypothetical protein SMU.1671c	Streptococcus mutans UA159
SDEG_0443	similar to putative ABC transporter, branched chain amino acid-binding protein	Streptococcus mutans UA159
SDEG_0565	similar to putative amino acid ABC transporter; integral membrane protein	Streptococcus mutans UA159
SDEG_1493	similar to putative ferrous ion transport protein A	Streptococcus mutans UA159
SDEG_1492	similar to putative ferrous ion transport protein B	Streptococcus mutans UA159
SDEG_0458	similar to putative methylated-DNA-protein-cysteineS-methyltransferase	Streptococcus mutans UA159
SDEG_1389		Streptococcus mutans UA159
SDEG_1529	probable ORF of C-terminal portion of hypothetical protein SMU.796	Streptococcus mutans UA159
SDEG_1530	probable ORF of N-terminal portion of hypothetical protein SMU.796	Streptococcus mutans UA159
SDEG_0718	pullulanase; similar to glycogen branching enzyme	Streptococcus mutansUA159
SDEG_0444	similar to putative branched chain amino acid ABC transporter, permease protein	Streptococcus mutansUA159
SDEG_0445	similar to putative branched chain amino acid ABC transporter, permease protein	Streptococcus mutansUA159
SDEG_1608	similar to hypothetical protein phiPH15 gp59	Streptococcus phage PH15
SDEG_0048	similar to vanZ protein putative	Streptococcus pneumoniae TIGR4
SDEG_0181	similar to MccC family protein	Streptococcus pneumoniae D39
SDEG_0207	similar to a putative protein kinase	Streptococcus pneumoniae TIGR4
SDEG_0208	similar to hypothetical protein SP 1060	Streptococcus pneumoniae TIGR4
SDEG_0209	similar to hypothetical protein SP 1059	Streptococcus pneumoniae TIGR4
SDEG_0210	similar to ABC transporter ATP-binding protein	Streptococcus pneumoniae TIGR4
SDEG_0211	similar to a putative ABC-2 transporter permease protein	Streptococcus pneumoniae TIGR4
SDEG_0514	similar to bacteriocin Blp	Streptococcus pneumoniae G54
SDEG_0588	similar to hypothetical protein spr1892	Streptococcus pneumoniae R6
SDEG_0590	similar to histidine kinase	Streptococcus pneumoniae R6
SDEG_0591	similar to phosphate ABC transporter, phosphate-binding protein	Streptococcus pneumoniaeSP9-BS68
SDEG_0593	similar to phosphate ABC transporter permease protein	Streptococcus pneumoniae TIGR4
SDEG_0595	similar to negative regulator of pho regulon forphosphate transport	Streptococcus pneumoniae R6
SDEG_0681	similar to PTS system IIC component	Streptococcus pneumoniae TIGR4
SDEG_0837	similar to a putative transcriptional regulator	Streptococcus pneumoniae TIGR4
SDEG_0841	similar to a putative transcriptional regulator	Streptococcus pneumoniae TIGR4
SDEG_0859	similar to homoserine kinase	Streptococcus pneumoniae D39
SDEG_0983	similar to transposase	Streptococcus pneumoniaeSP9-BS68
SDEG_1012	similar to hypothetical protein SPCG 0150	Streptococcus pneumoniae CGSP14
SDEG_1105	similar to hypothetical protein CGSSp14BS69 08645	Streptococcus pneumoniae SP14-BS69
SDEG_1158	similar to hypothetical protein CGSSp6BS73 08144	Streptococcus pneumoniae SP6-BS73
SDEG_1159	similar to hypothetical protein CGSSp6BS73 08139	Streptococcus pneumoniae SP6-BS73
SDEG_1334	similar to a putative transcriptional regulator PlcR	Streptococcus pneumoniae SP3-BS71
SDEG_1532	similar to transposase	Streptococcus pneumoniaeSP9-BS68
SDEG_1543	similar to transposase	Streptococcus pneumoniae SP9-BS68
SDEG_0384	similar to acetylornithine deacetylase/succinyl-diaminopimelate desuccinylase (M20/M25/M40/succinyl-diaminopimelate desuccinylase (M20/M25/M40family, acetylornithine deacetylase"	Streptococcus sanguinis SK36
SDEG_0186	similar to ATPase components of ABC transporters with duplicated ATPase domains, multidrug transport system	Streptococcus sanguinis SK36
SDEG_0196	similar to a putative bacteriocin ABC-type exporter ATP binding/permease protein	Streptococcus sanguinis SK36
SDEG_1592	similar to a putative histone acetyltransferase HPA2	Streptococcus sanguinis SK36
SDEG_0655	similar to hypothetical protein SSA 0868	Streptococcus sanguinis SK36
SDEG_1969	similar to hypothetical protein SSA 1981	Streptococcus sanguinis SK36
SDEG_1387	similar to a putative phosphoglycerate mutase family protein	Streptococcus sanguinis SK36
SDEG_1358	similar to immunoglobulin G-binding protein G precursor (IgG-binding protein G)	Streptococcus sp. G148
SDEG_0178	similar to hypothetical protein SsuiDRAFT 0887	Streptococcus suis 89/1591
SDEG_0184	similar to predicted transcriptional regulator	Streptococcus suis 98HAH33
SDEG_0189	similar to nucleotide-binding protein implicated ininhibition of septum formation	Streptococcus suis 98HAH33
SDEG_0212	similar to transposase IS204/IS1001/IS1096/IS1165	Streptococcus suis 89/1591
SDEG_0447	similar to ABC-type branched-chain amino acidtransport system ATPase component	Streptococcus suis98HAH33
SDEG_0471	similar to conserved hypothetical protein	Streptococcus suis 89/1591
SDEG_0623	similar to DNA-directed RNA polymerase specialized sigma subunit	Streptococcus suis 98HAH33
SDEG_0678	similar to transcriptional regulator	Streptococcus suis05ZYH33
SDEG_0679	similar to beta-galactosidase	Streptococcus suis 89/1591
SDEG_0682	similar to phosphotransferase system, mannose/fructose /N-acetylgalactosamine-specific component IID	Streptococcus suis 05ZYH33
SDEG_0683	similar to PTS system fructose subfamily IIA component	Streptococcus suis 89/1591
SDEG_0831	similar to ABC-type multidrug transport system ATPas and permease component	Streptococcus suis 98HAH33
SDEG_0858	similar to homoserine dehydrogenase	Streptococcus suis 89/1591
SDEG_0910	similar to dihydrofolate reductase	Streptococcus suis 98HAH33
SDEG_0937	similar to hypothetical protein	Streptococcus suis
SDEG_0971	similar to hypothetical protein SSU98 1762	Streptococcus suis 98HAH33
SDEG_0972	similar to 14-dihydroxy-2-naphthoate octaprenyltransferase	Streptococcus suis 05ZYH33
SDEG_0973	similar to membrane-associated lipoprotein involved in thiamine biosynthesis	Streptococcus suis 98HAH33
SDEG_0974	similar to hypothetical protein SSU05 1758	Streptococcus suis 05ZYH33
SDEG_1330	similar to ATPase component of ABC transporters with duplicated ATPase domains	Streptococcus suis 98HAH33
SDEG_1384	similar to haloacid dehalogenase-like hydrolase	Streptococcus suis 89/1591
SDEG_1400	similar to transcriptional antiterminator bglG	Streptococcus suis 89/1591
SDEG_1401	required for cellobiose uptake and metabolism	Streptococcus suis 89/1591
SDEG_1404	similar to hypothetical protein SsuiDRAFT 1295	Streptococcus suis 89/1591
SDEG_1406	similar to conserved hypothetical protein	Streptococcus suis 89/1591
SDEG_1414	PTS system beta-glucoside-specific IIABC component	Streptococcus suis 89/1591
SDEG_1429	similar to probable surface antigen negative regulator Par	Streptococcus suis 98HAH33
SDEG_1459	similar to superfamily I DNA and RNA helicase	Streptococcus suis 98HAH33
SDEG_1511	similar to hypothetical protein	Streptococcus suis

SDEG_1565	similar to glyoxalase/bleomycin resistance protein/dioxygenase	Streptococcus suis 89/1591
SDEG_1575	similar to predicted permease	Streptococcus suis 05ZYH33
SDEG_1599	similar to hypothetical protein SSU98 0605	Streptococcus suis 98HAH33
SDEG_1600	similar to transposase IS4	Streptococcus suis 89/1591
SDEG_1968	similar to lantibiotic efflux protein	Streptococcus suis 05ZYH33
SDEG_1970	similar to response regulator of the LytR/AlgRfamily	Streptococcus suis 98HAH33
SDEG_0251	similar to Crp-like transcriptional regulator	Streptococcus thermophilus LMD-9
SDEG_0292	similar to phage integrase familyintegrase/recombinase	Streptococcus thermophilus LMG18311
SDEG_0295	similar to lantibiotic biosynthesis protein	Streptococcus thermophilus LMG 18311
SDEG_0296	similar to lantibiotic efflux protein	Streptococcus thermophilus CNRZ1066
SDEG_0298	similar to predicted transcriptional regulator containing HTH domain	Streptococcus thermophilus LMD-9
SDEG_0446	similar to branched-chain amino acid ABC transporter ATP binding protein	Streptococcus thermophilus CNRZ1066
SDEG_0564	similar to aminoacid (glutamine) ABC transporter permease protein	Streptococcus thermophilus CNRZ1066
SDEG_0691	similar to glutathione S-transferase family	Streptococcus thermophilus LMG 18311
SDEG_0692	similar to adenosine deaminase	Streptococcus thermophilus LMG 18311
SDEG_0702	similar to phosphoserine phosphatase	Streptococcus thermophilus LMG 18311
SDEG_0743	associated with arginine deiminase pathway genes; probably functions in arginine catabolism	Streptococcus thermophilus LMG 18311
SDEG_0758	similar to hypothetical protein str1466	Streptococcus thermophilus CNRZ1066
SDEG_0759	similar to glycosyltransferase involved in cell wall biogenesis	Streptococcus thermophilus LMD-9
SDEG_0881	similar to hypothetical protein stu1191	Streptococcus thermophilus LMG 18311
SDEG_0968	similar to peptido glycan branched peptidesynthesis protein, putative alanine adding enzyme	Streptococcus thermophilus CNRZ1066
SDEG_0969	similar to peptido glycan branched peptidesynthesis protein, putative alanine adding enzyme	Streptococcus thermophilus CNRZ1066
SDEG_1013	similar to XRE family transcriptional regulator	Streptococcus thermophilus LMD-9
SDEG_1267	similar to hypothetical protein STER 0685	Streptococcus thermophilus LMD-9
SDEG_1405	similar to IS1191 transposase	Streptococcus thermophilus LMG 18311
SDEG_1898	similar to acetyltransferase GNAT family	Streptococcus thermophilus LMD-9
SDEG_1919	similar to hypothetical protein	Streptococcus thermophilus
SDEG_1573	similar to adhesion protein	Streptococcus uberis
Genes showing similarity to bacterial genes other than those of s*treptococci*		
SDEG_0079	similar to 50S ribosomal protein L14	Staphylococcus epidermidis ATCC 12228
SDEG_0086	similar to conserved hypothetical protein	Listeriamonocytogenes str. 4b H7858
SDEG_0182	similar to hypothetical protein PsycPRwf 0104	Psychrobacter sp. PRwf-1
SDEG_0187	similar to cyclic nucleotide-binding domain protein	Lactococcus lactis subsp. cremoris MG1363
SDEG_0188	similar to hypothetical protein llmg 1236	Lactococcus lactis subsp. cremoris MG1363
SDEG_0225	similar to glucanase; deblocking aminopeptidase	Bacillus cereus E33L
SDEG_0265	similar to conserved hypothetical protein	Enterococcus faecium DO
SDEG_0266	similar to hypothetical protein EF3281	Enterococcus faecalis V583
SDEG_0299	similar to 5-methylcytosine methyltransferase	Lactococcus lactis subsp. lactis bv. diacetylactis
SDEG_0300	similar to restriction enzyme	Lactococcus lactissubsp. lactis bv. diacetylactis
SDEG_0304	similar to hypothetical protein CLOSS21 00385	Clostridium sp. SS2/1
SDEG_0308	similar to hypothetical protein ANACAC 03037	anaerostipes caccae DSM 14662
SDEG_0309	similar to hypothetical protein ANACAC 03039	anaerostipes caccae DSM 14662
SDEG_0310	similar to hypothetical protein ANACAC 03038	anaerostipes caccae DSM 14662
SDEG_0311	similar to hypothetical protein CLOL250 01537	Clostridium sp. L2-50
SDEG_0330	similar to autolysin sensor kinase protein	Staphylococcus aureus subsp. aureus MRSA252
SDEG_0331	similar to two-component response regulator LytR	Carnobacterium sp. AT7
SDEG_0332	similar to murein hydrolase regulator LrgA	Staphylococcus aureus subsp. aureus JH9
SDEG_0333	in conjunction with LrgA this protein inhibits th eexpression or activity of extracellular murein hydrolases	Staphylococcus saprophyticus subsp. saprophyticus ATCC 15305
SDEG_0466	similar to L-lactate permease	Clostridium botulinum A str. ATCC 3502
SDEG_0690	similar to GCN5-related N-acetyltransferase	Actinobacillus succinogenes 130Z
SDEG_0755	similar to glycosyl transferase family protein	Anabaena variabilis ATCC29413
SDEG_0756	similar to glycosyl transferase	Leifsonia xylisubsp. xyli str. CTCB07
SDEG_0757	similar to rhamnosyltransferase	Leifsonia xylisubsp. xyli str. CTCB07
SDEG_0839	similar to hypothetical protein lin1204	Listeriainnocua Clip11262
SDEG_0931	similar to hypothetical protein CdifQCD-6 20778	Clostridium difficile QCD-63q42
SDEG_0953	similar to Type II restriction enzyme EcoRI	Parabacteroides distasonis ATCC 8503
SDEG_0954	similar to type II restriction enzyme EcoRI-likeprotein	Microcystis aeruginosa NIES-843
SDEG_0961	similar to ferric reductase	Oenococcus oeniPSU-1
SDEG_0962	similar to predicted ferric reductase	Bifidobacterium animalis subsp. lactis HN019
SDEG_0964	similar to oxidoreductase	Lactobacillus plantarumWCFS1
SDEG_0967	similar to hypothetical protein CAT7 10785	Carnobacterium sp. AT7
SDEG_1118	similar to putative endodeoxyribonuclease	Lactococcus phage r1t
SDEG_1197	similar to pyruvate oxidase or other thiaminepyrophosphate-requiring enzyme	Lactobacillus casei ATCC334
SDEG_1198	probable ORF of N-terminal portion of putativepyruvate oxidase	Lactobacillus paracasei
SDEG_1199	probable ORF of C-terminal portion of putativepyruvate oxidase	Lactobacillus paracasei
SDEG_1201	similar to phosphatase YbjI	Escherichia coli HS
SDEG_1204	similar to hypothetical protein RUMTOR 00521	Ruminococcus torques ATCC 27756
SDEG_1205	similar to permease	Campylobacter hominis ATCCBAA-381
SDEG_1206	similar to hypothetical protein CLORAM 03030	Clostridium ramosum DSM 1402
SDEG_1218	similar to SpiA (OrfY protein) (Putative sakacin Pimmunity protein)	Lactobacillus casei BL23
SDEG_1268	similar to hypothetical protein CLORAM 02318	Clostridium ramosum DSM 1402
SDEG_1291	similar to NADPH-dependent FMN reductase	Bacillus coagulans 36D1
SDEG_1318	similar to hypothetical protein ANACAC 02747	anaerostipes caccae DSM 14662
SDEG_1332	similar to ABC transporter ATP-bindingprotein/peptidase	Treponema denticola ATCC 35405
SDEG_1333	similar to hypothetical protein llmg 0328	Lactococcus lactis subsp. cremoris MG1363
SDEG_1339	similar to hypothetical protein PEPMIC 00190	Peptostreptococcus micros ATCC 33270
SDEG_1340	similar to hypothetical protein EUBDOL 02334	Eubacterium dolichum DSM 3991
SDEG_1341	similar to hypothetical protein PEPMIC 00192	Peptostreptococcus micros ATCC 33270
SDEG_1342	similar to hypothetical protein PEPMIC 00193	Peptostreptococcus micros ATCC 33270
SDEG_1343	similar to hypothetical protein PEPMIC 00194	Peptostreptococcus micros ATCC 33270
SDEG_1344	similar to hypothetical protein TDE2175	Treponemadenticola ATCC 35405
SDEG_1345	similar to a putative site-specific recombinase	Treponema denticola ATCC 35405
SDEG_1385	similar to hypothetical protein CLOBAR 01981	Clostridium bartlettii DSM 16795
SDEG_1390	similar to hypothetical protein lwe2314	Listeriawelshimeri serovar 6b str. SLCC5334
SDEG_1396	similar to hypothetical protein PEPE 0230	Pediococcus pentosaceus ATCC 25745
SDEG_1397	required for cellobiose uptake and metabolism	Bacillus coagulans 36D1
SDEG_1398	similar to conserved hypothetical protein	Bacillus coagulans 36D1
SDEG_1409	similar to putative phosphosugar-bindingtranscriptional regulator	Clostridium botulinum NCTC2916
SDEG_1411	similar to oxidoreductase	Lactobacillus plantarum WCFS1
SDEG_1437	similar to NADPH-dependent FMN reductase	Bacilluscoagulans 36D1
SDEG_1531	similar to hypothetical protein CLONEX 04238	Clostridium nexile DSM 1787
SDEG_1568	similar to hypothetical protein CLOBAR 02811	Clostridium bartlettii DSM 16795
SDEG_1569	similar to MOP/MATE family multidrug-resistanceefflux pump NorM	Fusobacterium nucleatum subsp.polymorphum ATCC 10953

SDEG_1574	similar to hypothetical protein LSL 1395	Lactobacillus salivarius UCC118
SDEG_1585	similar to alpha-1,2-mannosidase	Geobacillus sp.Y412MC10
SDEG_1595	similar to AMP-dependent synthetase and ligase	Rhodopseudomonas palustris BisA53
SDEG_1597	similar to putative acyl-CoA acetyltransferase FadA	Carnobacterium sp. AT7
SDEG_1598	similar to putative fatty oxidation complex protein	Staphylococcus saprophyticus subsp. saprophyticus ATCC15305
SDEG_1603	similar to hypothetical protein PEPMIC 00589	Peptostreptococcus micros ATCC 33270
SDEG_1606	similar to protein gp30	bacteriophage A118
SDEG_1607	similar to hypothetical protein PEPE 1706	Pediococcus pentosaceus ATCC 25745
SDEG_1618	similar to putative endodeoxyribonuclease	Lactococcus phage r1t
SDEG_1642	similar to Orf80	bacteriophage phi31.1
SDEG_1737	similar to conserved hypothetical protein	Listeriamonocytogenes str. 1/2a F6854
SDEG_1786	similar to transposase	Lactococcus lactis subsp.cremoris SK11
SDEG_1828	similar to hypothetical protein FN0115	Fusobacterium nucleatum subsp. nucleatum ATCC 25586
SDEG_1845	similar to rhodanese-like	Enterococcus faeciumDO
SDEG_1899	similar to alpha amylase catalytic region	Chloroflexus aurantiacus J-10-fl
SDEG_1900	similar to ABC-type sugar transport systemperiplasmic component	Lactococcus lactis subsp. cremorisSK11
SDEG_1901	similar to hypothetical protein llmg 1837	Lactococcus lactis subsp. cremoris MG1363
SDEG_1902	similar to ABC-type sugar transport system permease component	Lactococcus lactis subsp. cremoris SK11
SDEG_1903	similar to ABC-type polysaccharide transport system permease component	Lactococcus lactis subsp. cremorisSK11
SDEG_1904	similar to regulatory protein LacI	Clostridium beijerinckii NCIMB 8052
SDEG_1950	similar to hydrolase	Staphylococcus aureus RF122
SDEG_1981	similar to integral membrane protein	Lactobacillusplantarum WCFS1
SDEG_1986	similar to hypothetical protein llmg 0526	Lactococcus lactis subsp. cremoris MG1363
SDEG_1987	similar to hypothetical protein BIFDEN 02311	Bifidobacterium dentium ATCC 27678
SDEG_2005	similar to diacylglycerol kinase catalytic subunit	Enterococcus faecalis
SDEG_2023	similar to ABC transporter ATP-binding /membrane spanning permease - possible multidrug resistance	Carnobacterium sp. AT7
SDEG_2047	similar to amidohydrolase 2	Sphingomonas wittichii RW1
SDEG_2048	similar to amidohydrolase 2	Sphingomonas wittichii RW1
	No homolog in the databases	
SDEG_1601	no hit	
SDEG_1639	no hit	
SDEG_1643	no hit	
SDEG_2022	no hit	
SDEG_2024	no hit	
SDEG_2141	no hit	
SDEG_0180	no hit	
SDEG_0190	no hit	
SDEG_0301	no hit	
SDEG_0303	no hit	
SDEG_0378	no hit	
