# Supplementary material for: Complete genome sequencing and analysis of a Lancefield group G Streptococcus dysgalactiae subsp. equisimilis strain causing streptococcal toxic shock syndrome (STSS)
Source: BMC Genomics. 2011 Jan 11;12:17. doi: 10.1186/1471-2164-12-17 (PMC3027156; doi:10.1186/1471-2164-12-17)
Supplement: Additional file 5 — Features of CRISPR found in the GGS_124 genome and phages derived from GAS containing sequences homologous to GGS_124 spacers [file 1471-2164-12-17-S5.PDF]

Additional file 5. Features of CRISPR found in the GGS_124 genome and phages derived from GAS containing sequences homologous to GGS_124 spacers

CRISPR		Position in GGS_124 (length [bp])	Sequence	Phages* (virulence factors)	ID (bp/bp)	Position in subjects (length [bp])	Product#	Locus_tag#
Direct repeat		1173568-1174793 (1225)	GAATTGGGACCATTCAAAACAACATAGCTCTAAAAC					
Spacer	1	1173604-1173633 (30)	ACAGTTAAATAGCTTTGGTATCTTGTCGAT	phi3396(None)	25/25	37420-37444	Hypothetical protein	phi3396_62
	2	1173670-1173699 (30)	TCATCAAGGCTCTGCGCACCACCCAACGGA	Unknown				
	3	1173736-1173765 (30)	GCCTTAGGAAACAAAGCATTTACACTACCA	Unknown				
	4	1173802-1173831 (30)	AAAAGTGGCTATCACGCTGACACAGACTAA	370.1 (SpeC, MF2)	29/30	562397 - 562426	Hypothetical protein	SPy_0700
				phiMan.1 (MF3)	29/30	544754 - 544783	Hypothetical protein	SpyM50523
				phiMan.4 (MF)	29/30	1277388 - 1277417	Hypothetical protein	SpyM51272
				315.2 (SSA)	29/30	986086 - 986115	Hypothetical protein	SpyM3_0930
				315.4 (SpeK, Sla)	29/30	1240537 - 1240566	Hypothetical protein	SpyM3_1215
				8232.2 (SpeC)	29/30	611010 - 611039	Hypothetical protein	spyM18_0769
				9429.1 (SpeC, MF)	29/30	563274 - 563303	Phage endopeptidase	MGAS9429_ Spy0585
				10270.1 (SpeC, MF)	29/30	564551 - 564580	Phage endopeptidase	MGAS10270_ Spy0589
				10270.3 (SpeC)	29/30	1264948 - 1264977	Phage endopeptidase	MGAS10270_ Spy1311
				10750.1 (SpeC, MF)	29/30	581728 - 581757	Phage endopeptidase	MGAS10750_ Spy0614
				SPsP3 (SpeL)	29/30	656319 - 656348	Hypothetical protein	SPs0647
				phi3396 (None)	29/30	29056-29085	Phage endopeptidase	phi3396_47
				phiNIH1.1 (SpeC)	29/30	32615-32644	Hypothetical protein	30786..32930
				370.3 (SpeH, SpeI)	28/30	812835 - 812864	Hypothetical protein	SPy_0996
				8232.5 (Sda)	28/30	1460618 - 1460647	Pseudogene	spyM18_1760
				9429.2 (SpeA, SpeH)	28/30	810696 - 810725	Phage endopeptidase	MGAS9429_ Spy0842
				10394.3 (SpeK, Sla)	28/30	997762 - 997791	Phage endopeptidase	M6_Spy0994
				10394.7 (MF3)	28/30	1552427 - 1552456	Phage endopeptidase	M6_Spy1551
				SPsP5 (SpeC)	28/30	910779 - 910808	Hypothetical protein	SPs0926
				10394.1 (Sdn)	24/25	66749-66773	Phage endopeptidase	M6_Spy0058
				SPsP1 (Sdn)	24/25	477577-477601	Hypothetical protein	SPs0446
				315.6 (Sdn)	24/25	1419308-1419284	Hypothetical protein	SpyM3_1419
	5	1173868-1173897 (30)	AACACTGATTGTTCCAATTGTATTTGTAAA	Unknown				
	6	1173934-1173963 (30)	GTCACGCTGATATATGTCACCCAAATATAG	phi3396 (None)	30/30	35516-35487	Phage-associated cell wall hydrolase	phi3396_56
				10394.6 (Sda)	28/30	1546724 - 1546753	Phage-associated cell wall hydrolase	M6_Spy1544
	7	1174000-1174031 (32)	ACGCTTTAGCAGATGGAGCGGGGCTTACGAGG	315.4 (SpeK, Sla)	30/30	1262812 - 1262843	Hypothetical protein	SpyM3_1248
				6180.2 (SpeK, Sla)	30/30	1260125 - 1260156	Phage protein	M28_Spy126 6
				10270.3 (SpeC)	30/30	1287223 - 1287254	Phage protein	MGAS10270_ Spy1342
				SPsP3 (SpeL)	30/30	634042 - 634073		
				phiNIH1.1 (SpeC)	30/30	10338-10367		
				5005.1 (SpeA)	30/32	1011312 - 1011343	Phage protein	M5005_Spy_ 1034
				8232.5 (Sda)	30/32	1483150 - 1483181	Hypothetical protein	spyM18_1794
				NZ131.3 (MF3)	30/32	1492909 - 1492940	Hypothetical protein	Spy49_1499c
				phiMan.1 (MF3)	29/32	523159 - 523190	Putative phage endodeoxyribo nuclease	SpyM50489
	8	1174068-1174097 (30)	GCAAAAGCTTTCTCATATTTTACTCGATTC	Unknown				
	9	1174134-1174163 (30)	AGTATTCCAGTTCTCCATCAAGTAGTTCTC	9429.2 (SpeA, SpeH)	28/30	787932 - 787961	Phage protein RecT family	MGAS9429_ Spy0811
				370.3 (SpeH, SpeI)	25/25	788999 - 789028	Hypothetical protein	SPy_0958
	10	1174200-1174229 (30)	AATACAATCTCATGATTCGCAAACATCATG	Unknown				
	11	1174266-1174295 (30)	CATATTCTATTAATCCTTTATTTAAAAAAT	Unknown				
	12	1174332-1174361 (30)	GCAACAGTGTTTTCACGGTGGTTGCACGCC	Unknown				
	13	1174398-1174427 (30)	AAGCATCATAGTAAACATATCTAGCACGCT	8232.1 (SpeA)	30/30	316201 - 316230	Hypothetical protein	spyM18_0374
				phi3396 (None)	30/30	20016-19987	Hypothetical protein	phi3396_38
				315.1 (None)	29/30	771020 - 771049	Hypothetical protein	SpyM3_0715
				SPsP6 (None)	29/30	1125707 - 1125736	Hypothetical protein	SPs1137
	14	1174464-1174493 (30)	ATGCTCAAAAAGATGTGCTGTCCAACAGTA	Unknown				
	15	1174530-1174559 (30)	AAAGACGGTTTTTACTTTCAAAAATGAGAT	Unknown				
	16	1174596-1174625 (30)	ATGGTACAAGTAAAACTACCGCCCCAGTTA	Unknown				
	17	1174662-1174691 (30)	CAGACTTGGAATTGGCCAATGAGCGTGCCG	Unknown				
	18	1174728-1174757 (30)	TTCTGTCTAGCCCTTGTTCTTGACTCGTCT	Unknown				

MF, mitogenic factor; Sda, streptodornase alpha; Sdn, streptodornase; Sla, phospholipase A_2; Spe, streptococcal pyogenic exotoxin; SSA, streptococcal superantigen.

* Phages found in the GAS genome containing homologous spacer and virulence factors found in the corresponding phages are indicated.

# Putative gene products and their locus_tag containing the respective spacers are indicated.
