# Supplementary material for: Complete genome sequencing and analysis of a Lancefield group G Streptococcus dysgalactiae subsp. equisimilis strain causing streptococcal toxic shock syndrome (STSS)
Source: BMC Genomics. 2011 Jan 11;12:17. doi: 10.1186/1471-2164-12-17 (PMC3027156; doi:10.1186/1471-2164-12-17)
Supplement: Additional file 6 — Putative virulence factors found in GGS_124 and their homologous genes in other streptococcal species [file 1471-2164-12-17-S6.PDF]

Additional file 6. Putative virulence factors found in GGS_124 and their homologous genes in other streptococcal species

	S. dysgalactiae subsp. *equisimilis* GGS_124					Strain sharing homology with GGS_124			
**Locus tag**	**Product name**	**Gene**	**Signal peptide**	**LPXTG motif**	**% Identity**	**Strain**	**Product name**	**Locus tag/ Reference sequence**	**Gene**
				**Pore-forming toxins**					
SDEG_0427	Putative hemolysin		Signal peptide		95.05%	*S. pyogenes* MGAS315	HlyX, hemolysin	SpyM3_0276	*hlyX*
SDEG_0705	Streptolysin S precursor	*sagA*			88.68%	*S. pyogenes* MGAS315	Streptolysin S	SpyM3_0480	*sagA*
SDEG_0766	Pore forming protein	*ebsA*			88.27%	*S. pyogenes* MGAS315	Putative pore-forming peptide	SpyM3_0534	
SDEG_1015	HlyIII, hemolysin				92.59%	*S. pyogenes* MGAS315	HlyIII, hemolysin	SpyM3_0815	*hlyIII*
SDEG_1483	Hemolysin				98.91%	*S. pyogenes* MGAS315	HlyA1, hemolysin	SpyM3_1153	*hlyA1*
SDEG_2027	Streptolysin O precursor	*slo*	Signal peptide		98.77%	*S. pyogenes* MGAS315	Streptolysin O	SpyM3_0130	*slo*
				**Superantigens**					
SDEG_1991	Exotoxin G variant 4	*spegg4*			79.49%	*S. pyogenes* MGAS315	Streptococcal exotoxin SpeG	SpyM3_0155	*speG*
				**Proteases**					
SDEG_0233	Streptokinase		Signal peptide		88.18%	*S. pyogenes* MGAS315	Streptokinase A precursor	SpyM3_1698	*ska*
SDEG_0342	Membrane protease protein family				98.65%	*S. pyogenes* MGAS315	Hypothetical protein	SpyM3_1626	
SDEG_0468	Truncated cell envelope proteinase A	*cepA*	Signal peptide		62.82%	*S. pyogenes* MGAS315	ScpC/CepA/spyCEP	SpyM3_0298	*prtS*
SDEG_0574	Truncated cell-surface protease required for virulence cell		Signal peptide		49.36%	*S. agalactiae* A909	Cell surface serine endopeptidase CspA	SAK_1991	*cspA*
SDEG_0604	Putative peptidase				98.05%	*S. pyogenes* MGAS315	Peptidase U32 family small subunit (C1)	SpyM3_0417	
SDEG_0605	Peptidase family U32				98.13%	*S. pyogenes* MGAS315	Peptidase U32 family large subunit (C1)	SpyM3_0418	
SDEG_0673	Dipeptidase	*pepD*			98.92%	*S. pyogenes* MGAS315	Dipeptidase A	SpyM3_0465	*pepD*
SDEG_0933	Streptococcal C5a peptidase	*scpB*	Signal peptide	LPTTN	95.38%	*S. pyogenes* MGAS315	C5A peptidase precursor	SpyM3_1726	*scpA*
SDEG_1286	Putative exfoliative toxin				82.03%	*S. pyogenes* MGAS315	Putative exfoliative toxin	SpyM3_0632	
SDEG_1538	ATP-dependent endopeptidase		Signal peptide		83.19%	*S. pyogenes* MGAS315	ATP-dependent endopeptidase Lon	SpyM3_1187	
SDEG_1906	Putative C3-degrading proteinase				78.40%	*S. pyogenes* MGAS315	C3 degrading proteinase	SpyM3_1598	*cppA*
SDEG_2177	Endopeptidase	*htrA*			99.75%	*S. pyogenes* MGAS315	Putative serine protease	SpyM3_1864	*degP*
				**Adhesins**					
SDEG_0161	Fibronectin binding protein		Signal peptide	LPATG	62.30%	*S. pyogenes* MGAS10270	Fibronectin binding protein	MGAS10270_Spy0117	
SDEG_0230	Antiphagocytic M protein	*stg480.0*	Signal peptide	LPSTG					
SDEG_0237	Pullulanase		Signal peptide	LPQTG	79.95%	*S. pyogenes* MGAS315	Putative pullulanase	SpyM3_1694	*pulA*
SDEG_0704	Phosphopyruvate hydratase	*eno*			98.85%	*S. pyogenes* MGAS315	Enolase	SpyM3_0479	*eno*
SDEG_0935	Laminin binding protein	*lmb*	Signal peptide		99.67%	*S. pyogenes* MGAS315	Laminin binding protein	SpyM3_1725	*lmb*
SDEG_1263	Fibronectin binding protein	*fbp*			93.81%	*S. pyogenes* MGAS315	Fibronectin binding protein	SpyM3_0652	
SDEG_1358	Immunoglobulin G binding protein	*spg*	Signal peptide	LPTTG	81.62%	*S. pyogenes* MGAS315	Immunoglobulin G binding protein	CAA68489.1	*spg*
SDEG_1372	Internalin protein	*inlA*	Signal peptide		84.87%	*S. pyogenes* MGAS315	Putative internalin A precursor	SpyM3_1035	*inlA*
SDEG_1781	Putative collagen binding protein	*cbp*	Signal peptide	VPPTG	60.38%	*S. pyogenes* MGAS315	Putative collagen binding protein	SpyM3_0098	*cbp*
SDEG_1936	Glyceraldehyde-3-phosphate dehydrogenase, plasmin receptor	*gapA*			99.40%	*S. pyogenes* MGAS315	GAPDH plasmin receptor	SpyM3_0201	*plr*
SDEG_1984	Fibronectin binding protein		Signal peptide	LPATG	55.56%	*S. pyogenes* MGAS315	Fibronectin binding protein	SpyM3_0104	
				**Hyaluronidase/Hyaluronoglucosaminidase**					
SDEG_0654	Hyaluronate lyase precursor		Signal peptide		65.57%	*S. equi* subsp. *zooepidemicus* MGCS10565	Hyaluronate lyase precursor HylB	Sez_1299	*hylB*
SDEG_1588	Hyaluronoglucosaminidase	*hyl*			95.39%	*S. pyogenes* MGAS315	Putative hyaluronidase	SpyM3_1294	*hyl*
				**Metal transporters**					
SDEG_0431 to 0434	Ferrichrome transporter	*fhuGBDA*			86.72% to 91.39%	*S. pyogenes* MGAS315	FtsABCD, Ferrichrome transporter	SpyM3_0280 to 0283	*fhuGBDC*
SDEG_0488 to 0490	Metal transporter	*mtsABC*			86.72% to 91.18%	*S. pyogenes* MGAS315	MtsABC, metal transporter	SpyM3_0318 to 0320	*mtsABC*
SDEG_1860 to 1862	Ferrichrome transporter	*fhuBC*			77.12% to 83.82%	*S. pyogenes* MGAS315	HtsABC, Ferrichrome transporter	SpyM3_1558 to 1560	*fhuDGC*
				**Nucleases**					
SDEG_0541	Putative streptodornase		Signal peptide		59.64%	*S. pyogenes* M1T1	Phage-encoded extracellular streptodornase D Sda1	PHA01790	*sda1*
SDEG_0714	Extracellular nuclease		Signal peptide	LPKAG	73.33%	*S. pyogenes* SF370	Cell wall-located DNase SpnA	SPy_0747	
SDEG_0732	DNA-entry nuclease	*endA*	Signal peptide		55.12%	*S. pneumoniae* TIGR4	DNA-entry nuclease	SP_1964	
SDEG_0825	Putative cell surface 5'-nucleotidase		Signal peptide	LPMAG	75.96%	*S. pyogenes* MGAS315	5'-Nucleotidase	SpyM3_0591	
SDEG_1103	Deoxyribonuclease	*sdc*	Signal peptide		97.54	*S. pyogenes* MGAS315	Streptodornase (Sdn)	SpyM3_1409	*sdn*
				**Other chromosomal virulence factors**					
SDEG_0256	PTS system, IIB component		Signal peptide		98.94%	*S. pyogenes* MGAS315	Putative PTS system IIB component	SpyM3_1679	
SDEG_0322	Hypothetical membrane spanning protein		Signal peptide		76.13%	*S. pyogenes* MGAS2096	Hypothetical membrane spanning protein	MGAS2096_Spy1639	
SDEG_0327	Trigger factor	*ropA*			90.16%	*S. pyogenes* MGAS315	RopA, trigger factor	SpyM3_1634	*tig*
SDEG_0356	Oligopeptide binding protein	*oppA*	Signal peptide	IPFSG	96.34%	*S. pyogenes* MGAS315	Oligopeptide permease	SpyM3_0215	*oppA*
SDEG_0429	Putative manganese-dependent inorganic pyrophosphatase				96.14%	*S. pyogenes* MGAS315	Putative manganese-dependent inorganic pyrophosphatase	SpyM3_0278	
SDEG_0502	Surface antigen		Signal peptide		62.59%	*S. pyogenes* MGAS315	Hypothetical protein	SpyM3_0331	
SDEG_0503	67 kDa Myosin-crossreactive antigen				97.12%	*S. pyogenes* MGAS315	67 kDa Myosin-crossreactive streptococcal antigen	SpyM3_0332	
SDEG_0618	Hypothetical protein		Signal peptide		92.86%	*S. pyogenes* MGAS315	Hypothetical protein	SpyM3_0427	
SDEG_0619	Glutathione peroxidase				97.48%	*S. pyogenes* MGAS315	Putative glutathione peroxidase	SpyM3_0428	
SDEG_0725	ATP synthase B chain	*atpF*	Signal peptide		97.56%	*S. pyogenes* MGAS315	Putative proton-translocating ATPase subunit B	SpyM3_0495	
SDEG_0924	Periplasmic component of efflux system		Signal peptide		95.50%	*S. pyogenes* MGAS315	Putative ATP binding cassette transporter protein	SpyM3_1736	
SDEG_0929	Immunogenic secreted protein	*isp*	Signal peptide		96.13%	*S. pyogenes* MGAS315	Immunogenic secreted protein precursor	SpyM3_1731	*isp.1*
SDEG_1113	Collagen-like protein				41.20%	*S. equi* subsp. *equi* 4047	Collagen like protein	SEQ_0837	
SDEG_1216	Hypothetical membrane spanning protein		Signal peptide		93.81%	*S. pyogenes* MGAS315	Hypothetical protein	SpyM3_0740	
SDEG_1222	GTP binding protein	*lepA*			98.52%	*S. pyogenes* MGAS315	GTP binding protein LepA	SpyM3_0737	*lepA*
SDEG_1239	Hypothetical membrane associated protein				91.19%	*S. pyogenes* MGAS315	Hypothetical protein	SpyM3_0670	
SDEG_1259	Acid phosphatase, class B		Signal peptide	LPQAG	80.00%	*S. pyogenes* MGAS315	Acid phosphatase/phosphotransferase	SpyM3_0773	*aphA*
SDEG_1285	Hypothetical protein		Signal peptide		96.55%	*S. pyogenes* MGAS315	Hypothetical protein	SpyM3_0633	
SDEG_1309	D-alanine-poly(phosphoribitol) ligase subunit1	*dltA*			90.82%	*S. pyogenes* MGAS315	d-Alanine-d-alanyl carrier protein ligase	SpyM3_0994	*dltA*
SDEG_1542	Carbamate kinase	*arcC*	Signal peptide		99.37%	*S. pyogenes* MGAS315	Putative carbamate kinase	SpyM3_1191	*arcC*
SDEG_1548	Arginine deiminase	*arcA*			97.81%	*S. equi* subsp. *zooepidemicus* MGCS10565	Arginine deiminase ArcA	Sez_0568	*arcA*
SDEG_1555	Bifunctional methionine sulfoxide reductase A/Bprotein	*msrA*	Signal peptide		98.64%	*S. pyogenes* MGAS315	Putative methionine sulfoxide reductase	SpyM3_1267	*msrA.1*
SDEG_1581	Sugar binding protein		Signal peptide		97.09%	*S. pyogenes* MGAS315	Putative sugar ABC transporter substrate binding protein	SpyM3_1289	
SDEG_1698	Putative phosphohydrolase		Signal peptide		99.07%	*S. pyogenes* MGAS315	Hypothetical protein	SpyM3_1376	
SDEG_1865	Immunogenic secreted protein	*isp2*	Signal peptide		70.92%	*S. pyogenes* MGAS315	Immunogenic secreted protein precursor-like protein	SpyM3_1562	*isp.2*
SDEG_1935	Acid phosphatase	*lppC*	Signal peptide		82.39%	*S. pyogenes* MGAS315	Putative acid phosphatase	SpyM3_1625	*lppC*
SDEG_1940	Surface exclusion protein	*prgA*	Signal peptide		95.99%	*S. pyogenes* MGAS315	Putative surface exclusion protein	SpyM3_0197	*prgA*
SDEG_1980	UDP-glucose pyrophosphorylase	*hasC*	Signal peptide		98.32%	*S. pyogenes* MGAS315	HasC, HA capsule synthesis	SpyM3_0160	*hasC.2*
SDEG_2029	NADase	*nga*	Signal peptide		98.43%	*S. pyogenes* MGAS315	NAD glycohydrolase	SpyM3_0128	*nga*

Putative virulence factors were identified based on their homology to known bacterial virulence factors. Prediction of the signal peptide and the LPXTG motif were performed as described in Materials and Metho
