# Supplementary material for: Complete genome sequencing and analysis of a Lancefield group G Streptococcus dysgalactiae subsp. equisimilis strain causing streptococcal toxic shock syndrome (STSS)
Source: BMC Genomics. 2011 Jan 11;12:17. doi: 10.1186/1471-2164-12-17 (PMC3027156; doi:10.1186/1471-2164-12-17)
Supplement: Additional file 8 — Distribution of the virulence factors found in SDSE among other streptococci [file 1471-2164-12-17-S8.PDF]

Additional file 8. Distribution of the virulence factors found in SDSE among other *streptococci*

Virulence factors	Species (strain)						
	SDSE (GGS_124)	GAS (MGAS315)	SESE (4047)	SESZ (MGCS10565)	GBS (A909)	*S. uberis* (0140J)	Possible transmission mechanism*
HlyX	SDEG_0427	SpyM3_0276	SEQ_1844	Sez_1626	SAK_1432	SUB0416	transmitted vertically
HlyIII	SDEG_1015	SpyM3_0815	SEQ_1090	Sez_0959	SAK_1350	SUB0931	transmitted vertically
HlyA1	SDEG_1483	SpyM3_1153	SEQ_0643	Sez_0620	SAK_0600	SUB1273	transmitted vertically
streptolysin S	SDEG_0705	SpyM3_0480	SEQ_0546	Sez_0490	-	-	transmitted vertically
Streptolysin O	SDEG_2027	SpyM3_0130	-	-	-	-	transmitted vertically
NAD glycohydrolase	SDEG_2029	SpyM3_0128	-	-	-	-	transmitted vertically
SpeG	SDEG_1991	SpyM3_0155	-	-	-	-	transmitted vertically
SmeZ	-	M6_Spy1709	-	-	-	-	transmitted vertically
SSA	-	SpyM3_0920	-	-	-	-	horizontal transfer
SpeA	-	SpyM3_1301	-	-	-	-	horizontal transfer
SpeC	-	SPy_0711	-	-	-	-	horizontal transfer
SpeH	-	SPy_1008	2036	-	-	-	horizontal transfer
SpeI	-	SPy_1007	2037	-	-	-	horizontal transfer
SpeJ	-	SPy_0436	-	-	-	-	horizontal transfer
SpeK	-	SpyM3_1205	1728	-	-	-	horizontal transfer
SpeM	-	spyM18_1239	-	-	-	-	horizontal transfer
C3-degrading proteinase	SDEG_1906	SpyM3_1598	SEQ_0388	Sez_0324	SAK_1738	SUB1571	transmitted vertically
C5a peptidase	SDEG_0933	SpyM3_1726	SEQ_0563	Sez_0946	SAK_1320	SUB1154	transmitted vertically
Putatuve exfoliative toxin	SDEG_1286	SpyM3_0632	-	-	SAK_1301	SUB1136	transmitted vertically
Streptokinase	SDEG_0233	SpyM3_1698	-	-	-	SUB1785	transmitted vertically
Streptokinase (Skc)	-	-	SEQ_2014	Sez_1775	-	-	transmitted vertically
SpeB	-	SpyM3_1742	-	-	-	-	transmitted vertically
Laminin binding protein	SDEG_0935	SpyM3_1725	SEQ_1958	Sez_1736	SKA_1319	SUB0884	transmitted vertically
Pullulanase	SDEG_0237	SpyM3_1694	SEQ_0944	Sez_0820	SAK_1302	SUB0660	transmitted vertically
Collagen-like protein	SDEG_1113		SEQ_0837 / SEQ_0183	Sez_0729 / Sez_0608	SAK_0722	SUB1095 / -	transmitted vertically
Hyaluronan synthase (HasA)	-	SpyM3_1851	SEQ_0269	Sez_0199	-	SUB1697	transmitted vertically
glycosyl transferase, group 2 family protein	SDEG_0628	-	SEQ_0951	Sez_0827	SAK_1570	-	transmitted vertically
HasC (hasC.1/*hasC.2*)	SDEG_1980	SpyM3_1853 / SpyM3_160	SEQ_0271 / SEQ_0289	Sez_0201 / Sez_0216	SAK_0479	SUB1692/-	transmitted vertically
Hyaluronidase	SDEG_0654	SpyM3_665	-	Sez_1299	SAK_1284	-	transmitted vertically
DRS	SDEG_0932	MGAS2096_Spy1747	-	-	-	-	transmitted vertically

Based on the ontology analysis of the genome
