# Supplementary material for: Complete genome sequencing and analysis of a Lancefield group G Streptococcus dysgalactiae subsp. equisimilis strain causing streptococcal toxic shock syndrome (STSS)
Source: BMC Genomics. 2011 Jan 11;12:17. doi: 10.1186/1471-2164-12-17 (PMC3027156; doi:10.1186/1471-2164-12-17)
Supplement: Additional file 10 — List of oligonucleotide primers used in this study. [file 1471-2164-12-17-S10.PDF]

Additional file 10. List of oligonucleotide primers used in this study.

Primers for detection of CRISPRs and Cas genes

CRISPR/Cas	Primer	Sequence (5'-3')
CRISPR1 (detection and sequencing)	Forward	TCCAACTAACCCTCGCTCAACCCCATCCGG
CRISPR1 (detection and sequencing)	Reverse	TAGAAGCACGTGAACTATATGATTTTCCGC
CRISPR1 Cas genes (detection)	Forward	AAGACAATCAGTTCTGTAATCGTAGCAACC
CRISPR1 Cas genes (detection)	Reverse	TCAAACGGACAGCTCGTAGAAGATATACGC
CRISPR2 (detection and sequencing)	Forward	CCGAACCTTAGCCATCCTAACTCCTTTACC
CRISPR2 (detection and sequencing)	Reverse	AACACTTGGTCGCTCAGACAGCTATGCCCC
CRISPR2 Cas genes (detection)	Forward	GAACACGTTGCCCATAGTCCACACAGAG
CRISPR2 Cas genes (detection)	Reverse	TACCACGATCTAGGCAAAGCTGATCGAGCC
CRISPR1 in strain 164 (sequencing)	Reverse	CGCTTTCTGTTGAAAATCCTGTCACGAAA
CRISPR2 in strain 169 (sequencing)	Forward	TTTAGTCAGTTCATAGGTTACTCCATTGAT

Primers for detection of putative virulence factors unique to GGS_124

Locus tag	Primer name	Sequence (5'-3')
SDEG_0157	Forward	TCTTTTTTTAGAGACAGCTACGCCTGAA
SDEG_0157	Reverse	GTAAGTAATAAACTCCCGCAGGTAAGGA
SDEG_0158	Forward	ATGCAAAAATTATTGAAATACCTTTGTT
SDEG_0158	Reverse	TTACTTGTCCTCCCTGTGACGATCATGA
SDEG_0180	Forward	TGACAAAACGACAGCTCCGCTGGCTTCT
SDEG_0180	Reverse	AATAGACCAAATCTTGATGGTCTAGCAA
SDEG_0267	Forward	ATGACATCTATAAAAAAATCACTTGGAT
SDEG_0267	Reverse	TAAACCTGATAAAAGCCAAGGAAATCCT
SDEG_0574	Forward	GAACACAAAACAACGTTTTTCTCTCCGT
SDEG_0574	Reverse	TTAGTCCACAGCTTCTTTTAGGTCTGTA
SDEG_0805	Forward	TGCCAAAGGTCAAGCTTTATTTAGTCAA
SDEG_0805	Reverse	CATACCTTTAGCCTTATCCTTTTGTTTT
SDEG_0918	Forward	TGAACAAACGCATTAATGTAACGATAGT
SDEG_0918	Reverse	TATTCTTCTGGAACCGTTCTAAGATTGT
SDEG_0932	Forward	TGAAGATTAAAATTAGTAAAACACTACT
SDEG_0932	Reverse	AATACACAGCTTGTCCCCATTCAGAGGA
SDEG_0979	Forward	ATGAAACGGTATTCCAAAGGATTAATCA
SDEG_0979	Reverse	TTATTTTGTAACGTCTGATAGCCATACT
SDEG_1327	Forward	ATGAAAAAACTGTTGTCACTTGTTCTGA
SDEG_1327	Reverse	TTAGAAAGCAACCTTGGCATGACCGTTT
SDEG_1429	Forward	ATGAGACTACTGAAGGAAATCGATATTT
SDEG_1429	Reverse	TTAGTTGGCTCCTTCATCGAGGTAAATT
SDEG_1480	Forward	ATGTCTAATAAAAAAATATATTTCGCAA
SDEG_1480	Reverse	TCAGTCAATTGATGAGGGCTCAGTTTTT
SDEG_1511	Forward	CCGAGTTTGGCCGTTCGAAATGGAACGA
SDEG_1511	Reverse	TTTGGTGAATACGTCATCTTCGTTGATT
SDEG_1573	Forward	CTGAAAACAAAAAAGCCCAAGATGCACT
SDEG_1573	Reverse	TTAGCAGTTGCGCTATCGAGAGCGGCTT
SDEG_1601	Forward	ATGAAAAGTATCATCAAATTAGTTAGAA
SDEG_1601	Reverse	CTAATATGTCCTTCTGTCAGAACAAAGA
SDEG_1773	Forward	ATGAAAAATAAACTGATATTCGTGTTGT
SDEG_1773	Reverse	TTATTCTGTTTCCTTATTCTTTTTTGAA
SDEG_1969	Forward	ATGAAACGCATATTATTAGGAATTGGAT
SDEG_1969	Reverse	CTAAAGGTAATGAATCTCCATACTTGAA
SDEG_1984	Forward	AACCAGCTAATCTTCCTCTTTATGATAA
SDEG_1984	Reverse	ATCACATCTGGACCTTTGGTATCCTCAA
SDEG_2022	Forward	ATGAGGATCAAAAAAAGTTTAGTTATTT
SDEG_2022	Reverse	ATAAATGTTGTACAGAAAAGTGTTGCCT
SDEG_2141	Forward	ATGAAAAAGTTAGTCACTTTAGGAGCTA
SDEG_2141	Reverse	TCATTGCAAGTCTAATTCAACTACAAAT
FCT-6 like region (SDEG_0157 and 0158)	Forward	GTTGGTAGTAAGTTTCGATAATATCATGGC
FCT-6 like region (SDEG_0157 and 0158)	Reverse	TTGGTAATAAAGAAGCTGACTAGCGATGGG
